# Primate TNF Promoters Reveal Markers of Phylogeny and Evolution of Innate Immunity

**DOI:** 10.1371/journal.pone.0000621

**Published:** 2007-07-18

**Authors:** Andres Baena, Alan R. Mootnick, James V. Falvo, Alla V. Tsytsykova, Filipa Ligeiro, Ousmane M. Diop, Claudia Brieva, Pascal Gagneux, Stephen J. O'Brien, Oliver A. Ryder, Anne E. Goldfeld

**Affiliations:** 1 The CBR Institute for Biomedical Research, Harvard Medical School, Boston, Massachusetts, United States of America; 2 Gibbon Conservation Center, Santa Clarita, California, United States of America; 3 Laboratoire de Rétrovirologie, Institut Pasteur, Dakar, Senegal; 4 Unidad de Rescate y Rehabilitación de Animales Silvestres, Facultad de Medicina Veterinaria y Zootecnia, Universidad Nacional de Colombia, Bogotá, Colombia; 5 Project for Explaining the Origin of Humans, Glycobiology Research and Training Center, Department of Medicine and Cellular and Molecular Medicine, University of California at San Diego, La Jolla, California, United States of America; 6 Laboratory of Genomic Diversity, National Cancer Institute, Frederick, Maryland, United States of America; 7 Conservation and Research for Endangered Species, Zoological Society of San Diego, San Diego, California, United States of America; 8 Division of Biological Sciences, University of California at San Diego, La Jolla, California, United States of America; Sanofi-Aventis, United States of America

## Abstract

**Background:**

Tumor necrosis factor (TNF) is a critical cytokine in the immune response whose transcriptional activation is controlled by a proximal promoter region that is highly conserved in mammals and, in particular, primates. Specific single nucleotide polymorphisms (SNPs) upstream of the proximal human TNF promoter have been identified, which are markers of human ancestry.

**Methodology/Principal findings:**

Using a comparative genomics approach we show that certain fixed genetic differences in the TNF promoter serve as markers of primate speciation. We also demonstrate that distinct alleles of most human TNF promoter SNPs are identical to fixed nucleotides in primate TNF promoters. Furthermore, we identify fixed genetic differences within the proximal TNF promoters of Asian apes that do not occur in African ape or human TNF promoters. Strikingly, protein-DNA binding assays and gene reporter assays comparing these Asian ape TNF promoters to African ape and human TNF promoters demonstrate that, unlike the fixed differences that we define that are associated with primate phylogeny, these Asian ape-specific fixed differences impair transcription factor binding at an Sp1 site and decrease TNF transcription induced by bacterial stimulation of macrophages.

**Conclusions/significance:**

Here, we have presented the broadest interspecies comparison of a regulatory region of an innate immune response gene to date. We have characterized nucleotide positions in Asian ape TNF promoters that underlie functional changes in cell type- and stimulus-specific activation of the TNF gene. We have also identified ancestral TNF promoter nucleotide states in the primate lineage that correspond to human SNP alleles. These findings may reflect evolution of Asian and African apes under a distinct set of infectious disease pressures involving the innate immune response and TNF.

## Introduction

**Figure 1:**
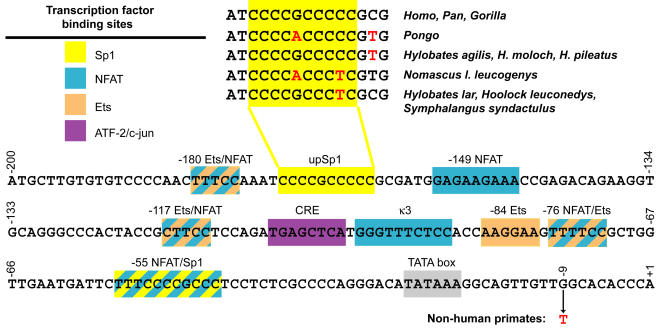
The human TNF gene promoter. The sequence of the human TNF promoter from −200 to +1 nt relative to the start site of transcription is shown, along with previously identified phylogenetic footprints [9] and binding sites for the indicatedtranscription factors, including sites identified as binding sites for multiple factors (crosshatched) in quantitative DNase I footprinting assays, by EMSA analysis, and by chromatin immunoprecipitation assays [1]–[7]. The sequence of the upstream Sp1 site (upSp1) found in the indicated genera and species is shown, with fixed nucleotide differences from the human sequence indicated in red. The thymine at position −9, which is invariant in all non-human primates studied previously [9] and in this study, is also shown in red.

The gene encoding tumor necrosis factor (TNF), a cytokine that plays a critical role in lymphocyte biology and in the innate and adaptive immune response, is transcribed rapidly and to high levels in response to multiple stimuli. This process is directed by the formation of inducer-specific nucleoprotein complexes, known as enhanceosomes, over a region of approximately 200 base pairs upstream of the start site of transcription [1]–[4]. The non-coding DNA elements within the TNF promoter that control stimulus-specific transcription of the TNF gene, and the recruitment of their cognate transcription factors, have been extensively characterized [1]–[7], which provides an opportunity to examine these functional regions across closely related species. Indeed, in an earlier study, sequence comparisons among representative primate species, including New World monkeys, revealed regions of complete sequence conservation, or “phylogenetic footprints [8],” from positions −131 to −63 and −53 and −45 relative to the start site of TNF gene transcription (Figure 1), in the core region of the enhanceosome [9]. By contrast, sequences outside of transcriptionally important regions showed high variability [9].

The human TNF gene is located within the most polymorphic region of the human genome, the major histocompatibility complex (MHC) region of chromosome 6, which includes the human leukocyte antigen (HLA) genes [10]. At least ten common single nucleotide polymorphisms (SNPs) have been identified in the human TNF promoter, all of which fall outside of the identified TNF primate phylogenetic footprints [11]–[19], a feature consistent with the accrual of mutations under neutral evolution [9], [20]. In fact, several of the these TNF promoter SNPs have been shown to be in non-random association with extended HLA haplotypes [16], [17], [19], [21], which are conserved blocks of DNA sequences at the MHC locus between HLA-B and HLA-DR on chromosome 6, where the TNF gene is located [22], [23].

Since TNF promoter SNPs were first described, multiple laboratories have studied their association with susceptibility to autoimmune and infectious diseases. Although statistical associations with a variety of diseases have been found in some studies, there has been no consistent functional role demonstrated for any TNF promoter SNP in the regulation of the TNF gene itself [24]–[28]. Rather, these SNPs have been shown to be markers of human ethnic diversity [19] and are likely markers for HLA or for other genes yet to be identified that are involved in disease susceptibility in different human ethnic groups [17], [19]. By contrast, through detailed examination of 411 TNF promoters from individuals of distinct ethnic backgrounds, we demonstrated that the 200 bp proximal promoter involved in the regulation of the gene is completely conserved in humans, and defined the canonical human TNF promoter sequence up to approximately 1.2 kb relative to the transcription start site [17], [19], [29].

**Figure 2:**
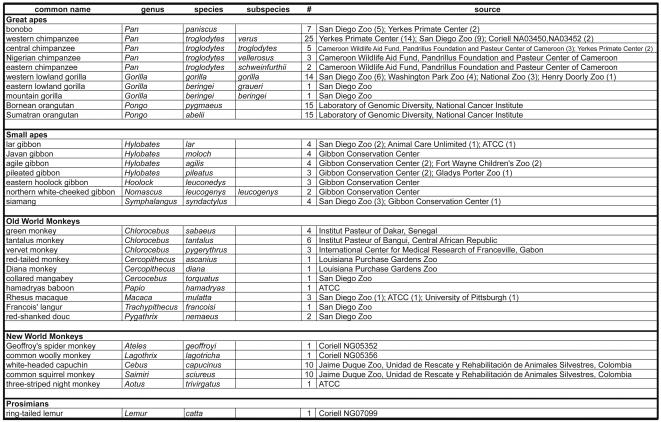
Primate species examined in this study. The common and taxonomic designation for each species or subspecies [110], [111], number of individuals from which DNA for sequence analysis was isolated, and the source of the sampled individuals are indicated. The region of the promoter corresponding to the upstream Sp1 site (Figure 1) was sequenced for *Cercopithecus ascanius, Trachypithecus francoisi, Pygathrix nemaeus*, and *Lemur catta* (Figure 7A). For all other species and subspecies, the complete proximal and distal TNF promoter regions (−1153 to +69 nt relative to the start site of transcription) were sequenced.

Here, we have undertaken one of the most broadly inclusive analyses to date of a mammalian gene regulatory region, as well as the broadest examination of the promoter region of an innate immune response gene, across primate species. Specifically, we isolated and sequenced approximately 1.2 kb of the upstream promoter region of the TNF gene from 29 non-human primate species and subspecies. These included multiple individuals representing all species of the great apes [bonobo (*Pan paniscus*), chimpanzee (*Pan troglodytes*), gorilla (*Gorilla*), and orangutan (*Pongo*)], all four genera of gibbon (Hylobatidae), and multiple species of Old World monkeys (OWM) and New World monkeys (NWM) (Figure 2).

Using a comparative genomics approach, we found a total of 332 fixed differences from the consensus human TNF promoter sequence in the primate species examined. We show that 41 of these fixed genetic differences serve as markers of primate species and subspecies. Strikingly, the monoallelic or biallelic variants carried in multiple human TNF promoter SNPs match divergent sequences in the primate lineage. In fact, the rare alleles found in two human SNPs (−1030 T/C and −375 G/A) are identical to TNF promoter sequences found at the same position in the New World monkey clade, which diverged from the common ancestor of other primates roughly 35 million years ago.

We also identified fixed differences unique to TNF promoters from the Asian apes in an Sp1 site that lies −173 to −163 nt relative to the start site of transcription of the human TNF promoter. We have previously shown that this Sp1 site is involved in the regulation of the human TNF gene in macrophages in response to stimulation with lipopolysaccharide (LPS), a cell wall component of gram-negative bacteria, or to *Mycobacterium tuberculosis* (MTb) stimulation, but that this site is not required for human TNF gene regulation in T cells stimulated via antigen receptor engagement or calcium influx [3], [4]. These data are consistent with the cell type- and inducer-specific regulation of the TNF gene [1]–[7].

Remarkably, here we show that these Asian ape-specific fixed genetic differences impair binding of Sp1 and Sp3 proteins to the site and decrease the relative level of TNF gene transcription in response to LPS and MTb as compared to the human or African ape TNF promoters. By contrast, these changes have no impact on the regulation of the gene in T cells by calcium influx, consistent with previous studies of the effects of specific mutations in this site in stimulus-specific regulation of the human TNF gene promoter. Thus, we provide evidence for adaptive changes in an innate immune response gene correlated with the current geographic distribution of higher primates. Furthermore, we demonstrate that fixed nucleotide differences and polymorphisms in the TNF promoter serve as markers of primate ancestry and biodiversity.

## Results

### Fixed differences in the TNF promoter mark primate phylogeny

We obtained blood, cell lines, or DNA samples from multiple animals representing African and Asian ape species and subspecies. Specifically, we obtained samples from the bonobo (*Pan paniscus*); the chimpanzee (*Pan troglodytes*), including the four subspecies of chimpanzee; the two species of gorilla, the western (*Gorilla gorilla*) and the eastern (*Gorilla beringei*) gorilla; the two species of orangutan, the Bornean (*Pongo pygmaeus*) and the Sumatran (*Pongo abelii*) orangutan; and seven gibbon species representing all four genera of gibbon [the lar gibbon *(Hylobates lar)*, the agile gibbon *(Hylobates agilis)*, the Javan gibbon *(Hylobates moloch)*, the pileated gibbon *(Hylobates pileatus)*, the eastern hoolock gibbon *(Hoolock leuconedys)*, the siamang *(Symphalangus syndactylus)*, and the northern white-cheeked gibbon *(Nomascus l. leucogenys)*]. We also obtained samples from several species of African and Asian OWM and NWM (Figure 2).

**Figure 3:**
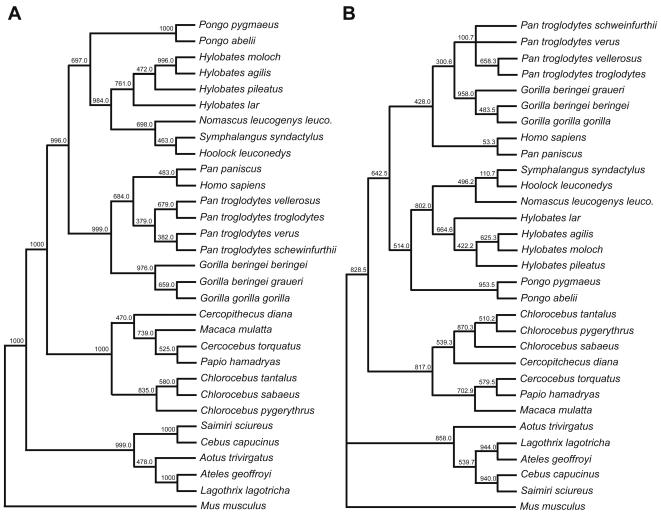
TNF promoter-based primate phylogenetic trees. Extended majority rule consensus trees derived from 1000 replications by bootstrap analysis generated from a distance matrix between ∼1.2 kb of sequence for each taxon using the Kimura 2-parameter model (A) and maximum parsimony (B) is shown. Values at internodes indicate the number of times the group consisting of the species to the right of the internode occurred among the trees (out of 1000 trees).

Approximately 1.2 kb of the TNF promoter was sequenced for each sample, and the sequences corresponding to positions −1153 to +69 nt relative to the start site of transcription in the human TNF promoter were aligned (Figure S1) and analyzed. Using the mouse TNF promoter sequence as the outgroup, we performed phylogenetic analyses and obtained very strong bootstrap values for the nodes of divergence of the genera using neighbor joining methods based on genetic distances (Kimura 2-parameter model) as well as maximum parsimony (Figure 3). The species fell into clear OWM (*Chlorocebus, Cercopithecus, Cercocebus, Papio, Macaca*) and NWM (*Ateles, Lagothrix, Cebus, Saimiri, Aotus*) clades, similar to the OWM and NWM groups observed in the analysis of genetic distances based on the promoter or open reading frame of the CC chemokine receptor 5 (CCR5) gene [30]. It is interesting to note that the coding region of the TNF gene, which exhibits up to 99% nucleotide sequence homology between humans and primate species of the *Papio, Macaca, Cercocebus, Saimiri*, and *Aotus* genera [31]–[35] exhibited *Papio*-*Macaca* and *Saimiri*-*Aotus* clades in a Kimura 2-parameter analysis of genetic distances [35] consistent with the OWM and NWM split we observe with the 5′ non-coding regions.

In general, the majority of gene sequences previously analyzed in hominids supports a chimpanzee-human clade, although a smaller proportion of the analyzed genes is consistent with a chimpanzee-gorilla or human-gorilla clade [36]–[40]. Interestingly, in the case of the TNF promoter, both approaches to phylogenetic inference that we utilized (neighbor joining and maximum parsimony) yield a bonobo-human clade, as well as a chimpanzee-gorilla clade (Figure 3). Similarly, our phylogenetic analyses yield a gibbon-orangutan clade, while evolutionary relationships place orangutans in a hominid clade with great apes and humans [41]–[43]. These observations indicate that sequence conservation among primate TNF promoters can reflect homoplasy or lineage sorting inconsistent with the species tree due to short intervals between subsequent speciation events, a point that is important to consider in the context of human TNF promoter SNP variant alleles, as will be discussed below.

We identified 332 fixed genetic differences (single nucleotide changes, insertions, and deletions) in these primate TNF promoters relative to the human sequence (Figure S1 and Table S1). The fixed genetic differences are homozygous and invariant in all representative individuals from a species or subspecies. A total of 33 (∼10%) fixed differences lie within 200 bp upstream of the start site of transcription, that is, within the proximal core TNF promoter region required for transcriptional activation of the TNF gene in response to multiple stimuli. Of these, only 5 are found in apes and only one occurs in the African apes: a G/T transversion at position −9 [9], which is completely conserved in all non-human primates examined (Figure S1 and Table S1). Notably, the phylogenetic footprints from positions −131 to −63 and −53 and −45 relative to the start site of TNF gene transcription [9] are interrupted only by the −89 C/T transition in the vervet monkey (*Chlorocebus pygerythrus*) and the −98 T/A transversion in the white-headed capuchin (*Cebus capucinus*; Figure S1). We also identified 16 SNPs within specific primate species from apes, OWM, and NWM representatives examined (Table S2).

**Figure 4:**
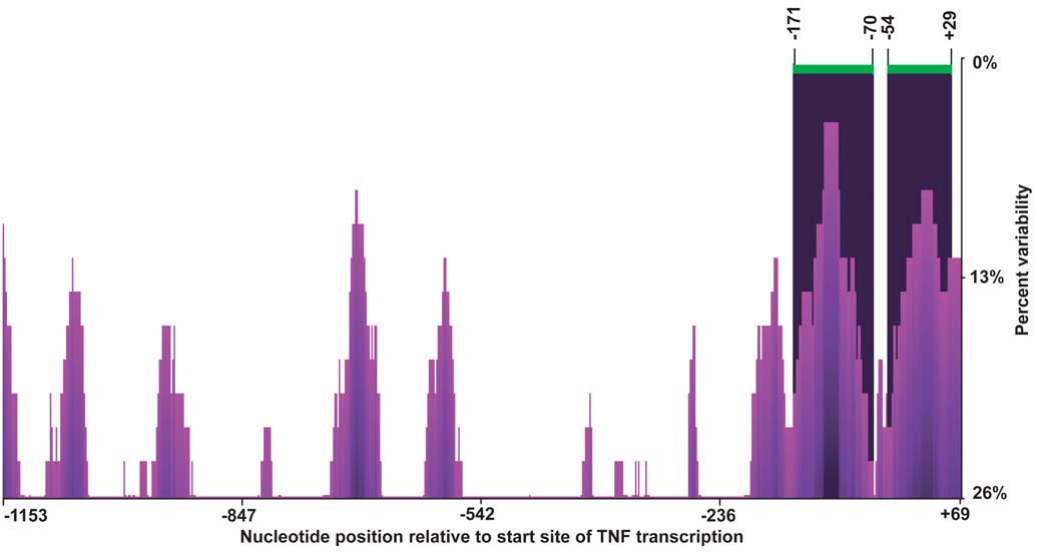
Conservation of the proximal TNF promoter region in primates. Multiple sequence alignment (MSA) of the TNF promoter (−1153 to +69 nt relative to the start site of transcription) for human and all other primate species examined in this study using eShadow [47]. Nucleotide position relative to the TNF start site of transcription is on the x-axis. Percentage variation (inversely proportional to conservation) is on the y-axis. Peaks and valleys of the conservation plot correspond to regions of low and high variation, respectively, and 0% variation signifies 100% sequence identity in the MSA. Black shading indicates regions of highest sequence conservation.

Functional elements in mammalian gene promoters can be identified by high conservation between species distantly related on the evolutionary scale, particularly for genes with conserved function across different taxa [44], [45] . The proximal TNF promoter shows high sequence conservation among primates, with complete conservation within phylogenetic footprints across seven primate species, including OWM and NWM [9]. Sequence divergence among primate genomes is inherently low since primates are relatively closely related on the evolutionary scale among vertebrates. However, comparisons between gene sequences in primates can be considerably improved through the technique of phylogenetic shadowing [46], [47]. In order to compensate for the relatively low level of sequence variation among closely related species, this technique takes into account the phylogenetic relationships among the set of species to be analyzed and the rate at which mutations would be expected to accumulate over time. Thus, rather than directly assessing the degree of conservation among sequences, phylogenetic shadowing reveals regions that have accumulated sequence variation at a relatively slower rate, indicative of conserved function [46]. This can be visualized as a conservation plot of the likelihood ratio that a given nucleotide position experienced relatively faster or slower accumulation of variation over time; the strongest peaks of the conservation plot, that is, regions of low variation, are expected to correlate with functional regions [47]. As shown in Figure 4, when all of the primate TNF promoter sequences from this study are analyzed by phylogenetic shadowing, the regions of lowest variation lie within the proximal 200 base pairs involved in gene activation and enhanceosome formation. These regions of low variation also coincide with elements identified as functional binding sites for transcription factors, which lie within the proximal promoter (Figure 1). Although putative binding sites for the NF-κB transcription factor lie outside of the proximal TNF promoter, these do not coincide with the peaks in Figure 4, nor are they conserved between human and mouse [48], consistent with our previous studies demonstrating that the proximal ∼200 base pairs of the TNF promoter are sufficient for directing activation of TNF gene transcription in response to multiple stimuli [1]–[4], [6], [48]. Thus, this analysis, which takes into account the evolutionary relationships among the multiple primate species and subspecies studied here, strongly corroborates the conclusion [9] that the proximal TNF promoter is a highly conserved functional element.

### Fixed differences in the TNF promoter distinguish primate species and subspecies

**Figure 5:**
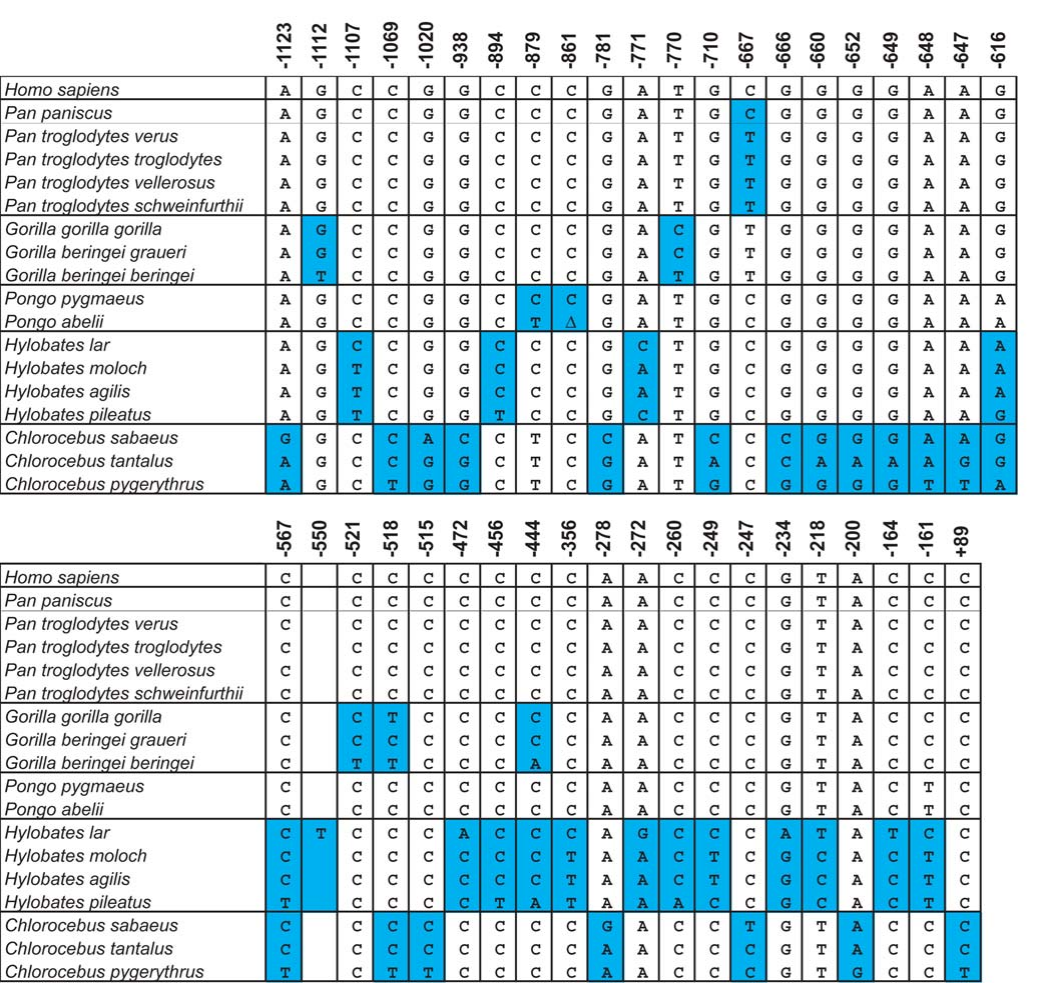
Fixed differences in the TNF promoter distinguish primate species and subspecies. Column headers indicate the position in the human TNF promoter relative to the start site of transcription. The corresponding nucleotide sequence, deletion, or insertion for each species is indicated for each row. Blue shading in a column indicates groups of primate subspecies within a species (or species within a genus) distinguished by a fixed difference.

In the primate species examined in this study, there are a total of 332 fixed differences between the positions corresponding to −1153 to +69 of the human TNF promoter region. Notably, we found that 41 of these fixed differences can be used to distinguish species and subspecies in primates, including the great apes (Figure 5). For example, two fixed TNF promoter genetic differences distinguish *Pongo pygmaeus* from *Pongo abelii*: the cytosine residues present at positions −879 and −861 in *Pongo pygmaeus* and humans are a thymine and a single nucleotide deletion, respectively, in *Pongo abelii*. In gorillas, five fixed differences at positions −1112, −770, −521, −518, and −444 distinguish three subspecies within the genus *Gorilla*: the western lowland gorilla *(G. g. gorilla)* is marked by −770 T/C and −518 C/T; the eastern lowland gorilla *(G. beringei graueri)*, by −770 T/C; and the mountain gorilla *(G. b. beringei)*, by −1112 G/T, −521 C/T, −518 C/T, and −444 C/A. We note that we only have one representative of *G. beringei graueri* and *G. b. beringei* because they are critically endangered species; thus, we formally cannot rule out that these sequence differences represent SNPs. A number of SNPs were identified for *Pan troglodytes*, but no fixed differences occurred among the four chimpanzee subspecies (Table S2). All four subspecies of *Pan troglodytes* do contain a shared species-specific fixed difference, however: a thymine at position −667, where a cytosine occurs in *Pan paniscus* and humans.

### Multiple human TNF SNP variant alleles correspond to divergent sequences in primates

**Figure 6:**
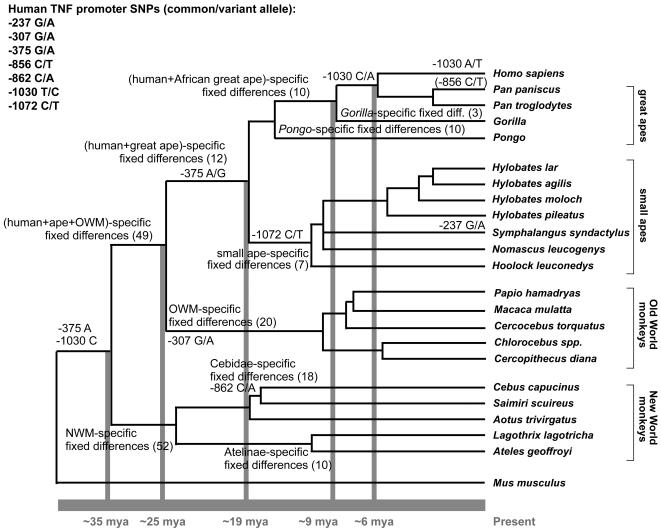
Fixed differences in the TNF promoter mark primate ancestry. Clade diagram illustrating the basic and approximate evolutionary relationships among the primate species in this study, with selected times of divergence (in millions of years) noted. Groups of fixed genetic differences (the total number in parenthesis) completely conserved within clades are marked at the appropriate branch points (see Table S3). Human TNF promoter SNPs which are ancestral in the primate lineage are listed. Ancestral sequences and transitions or transversions in the TNF promoter are noted at the appropriate branchpoints.

Placing comparative sequence analysis of the TNF promoter from multiple primate species in the context of known primate phylogeny demonstrates that specific single nucleotide positions are characteristic of different primate taxa (Figure 6). That is, certain fixed genetic differences identified in this study are shared between humans and other primate clades. For example, some fixed genetic differences are shared by species within the African great ape clade, within the ape clade, or within the clade encompassing all apes and OWM (Table S3A), while others are completely conserved only within specific primate clades, for example, NWM, OWM, gibbons, orangutans, and gorillas (Table S3B). By placing these fixed differences in the context of evolutionary relationships among primates, the ancestral nucleotide state of these fixed differences can be deduced and we can estimate the point at which a given fixed nucleotide difference diverged among the primate taxa (Figure 6). Furthermore, it allows us to assess which alleles of human TNF promoter SNPs at these positions are identical to divergent sequences in the primate lineage, indicating that a given allele in the human population has arisen either directly (identical by descent) or independently (identical by state) from primate ancestors. Strikingly, we found that distinct alleles of a total of six human TNF promoter SNPs (−237 G/A, −307 G/A, −375 G/A, −862 C/A, −1030 T/C, and −1072 C/T) are identical to an ancestral nucleotide in the primate lineage, and that a seventh human SNP, −856 C/T, is also a SNP in *Pan paniscus*.

As shown in Figure 6, the rare alleles of four human TNF promoter SNPs (−307 G/A, −862 C/A, −1072 C/T, and −237 G/A) were detected as fixed sequence differences unique to specific primate taxa. The rare allele of the −307 G/A human SNP, which has been identified as a fixed nucleotide in some species of Old World monkeys [9], [49], is present in all OWM species studied here. This indicates that the adenine at position −307 became fixed in the Cercopithecoidea superfamily (OWM) at the point at which it diverged from the Hominoidea superfamily (apes and humans) roughly 23–25 million years ago [39], [41]–[43], [50], [51], approximately at the geologic boundary between the Oligocene and Miocene (23.3 million years ago) [52]. Similarly, we detected the rare allele of the −862 C/A human SNP as a fixed nucleotide in NWM of the Cebidae family, *Cebus capucinus* and the common squirrel monkey (*Saimiri sciureus*), indicating that the fixation of the adenine at position −862 correlates with the divergence of the Cebidae from common primate ancestors of the NWM approximately 16–20 million years ago [53], [54].

The rare allele of the −1072 C/T human SNP is present in all gibbon species examined here, indicating that the thymine at position −1072 was fixed in the gibbon lineage at a time corresponding to the point of origin of the Hylobatidae, which is roughly 15–19 million years ago [37], [42], [43], [50], [55]. Within the Hylobatidae, we detected the rare allele of an additional human SNP, −237 G/A, as a fixed nucleotide difference only in *Symphalangus syndactylus*. Four clades of gibbons, recently designated as genera and distinguished by distinct diploid chromosome number, are well established: *Hylobates, Hoolock, Symphalangus*, and *Nomascus* [56], [57]. Although most studies conclude that *Hylobates* diverged most recently, comparative genomics analysis of nuclear and mitochondrial DNA sequences has yielded conflicting models of the phylogenetic relationships among the genera (for example, see [58]–[60]) hindered by the unusually rapid chromosome rearrangement rate in gibbons [61]. Thus, fixation of the adenine at position −237 in *Symphalangus* likely dates to a point in gibbon evolution after the fixation of the thymine at position −1072 and is a derived condition in that clade.

In contrast to the other human TNF promoter SNPs described above, the rare alleles of the −375 G/A and −1030 T/C human SNPs, rather than the common alleles, correspond to ancestral nucleotide states in the primate lineage. As shown in Figure 6, while the rare allele of the −307 G/A SNP became fixed in OWM following their divergence from other primates ∼25 million years ago, the common allele of the −375 G/A SNP became fixed in the rest of the Hominoidea superfamily (gibbons, great apes, and humans), while the rare allele is present in all OWM and NWM.

In the case of the −1030 T/C human SNP, all primates except for the Hominini tribe (the genus *Pan* and the genus *Homo*) bear a cytosine, the rare allele in humans, as a fixed difference at position −1030 in the TNF promoter, while *Pan pansicus* and *Pan troglodytes* bear an adenine at position −1030. Thus, the *Pan*-specific cytosine to adenine transversion at position −1030 appears to coincide with the divergence between the human-chimpanzee clade and gorilla clade approximately 6–9 million years ago [37], [39], [42], [43], [50], [51], while the human-specific shift to thymine appears to coincide with the human-chimpanzee split 5–7 million years ago [37], [39], [41]–[43], [50], [51], [55], [62] (Figure 6).

Notably, the sequence context of the ancestral cytosine at position −1030 in the TNF promoter places it in a CpG dinucleotide, which is particularly prone to mutation, as has been observed in comparisons between the chimpanzee and human genomes [63]–[66]. It is also interesting to note that in humans, the −1030 T/C SNP occurs in linkage with the −862 C/A SNP [16], [19], [29], [67]–[69]. These linked −1030/−862 SNPs have been shown to be in strong linkage with the B35 HLA haplotype, which is associated with HIV susceptibility. Consistent with this, these SNPs are thus associated with lack of control of HIV-1 viremia [29], most likely due to this linkage [70]. Taken together these associations suggest that the common thymine at the position in humans was positively selected in a cassette of closely spaced genes.

Finally, one human TNF promoter SNP, −856 C/T, is also polymorphic in *Pan paniscus*, an unusual event since SNPs are shared among great ape species at the expected neutral rate. For example, a study of over 200 orthologues of human SNP sites in *Pan paniscus, Pan troglodytes*, and *Gorilla gorilla* revealed only three shared polymorphisms between humans and *Pan pansicus* and two shared polymorphisms between humans and *Gorilla gorilla*; like the −856 C/T SNP, all of these *Pan paniscus* SNPs (and one of the two *Gorilla gorilla* SNPs) involved a CpG dinucleotide [63]. Such shared polymorphisms may arise independently, which may be evidence for stabilizing selection to maintain the SNP, or they may be identical by descent [63]. Notably, this SNP is highly expressed in human Amerindian populations, up to 45% in the Paez, and is in linkage with Amerindian HLA haplotypes [19], suggesting that it is a marker for a cassette of genes that are positively selected in humans.

### Fixed differences between Asian and African apes affect stimulus-specific TNF gene activation

**Figure 7:**
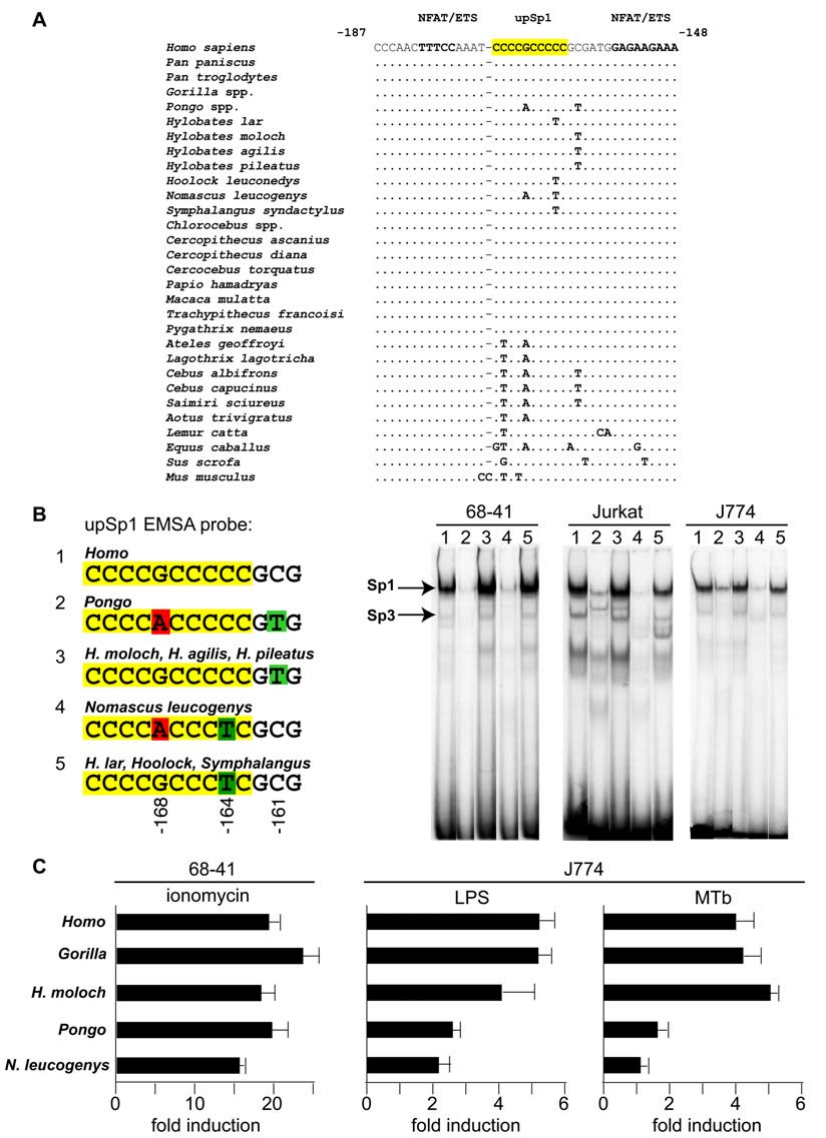
Functional analysis of fixed genetic differences in the upstream Sp1 site in primate TNF promoters. A. Sequence of the upstream Sp1 site in the TNF promoter from humans and the indicated non-human primates, with the horse (*Equus caballus*) and pig (*Sus scrofa*) sequences for comparison. Nucleotide positions relative to the start site of TNF transcription are shown. B. EMSA of nuclear extracts from the indicated cell lines incubated with radiolabeled oligonucleotides corresponding to the indicated primate TNF upstream Sp1 site. Positions of the Sp1- and Sp3-DNA complexes, confirmed by antibody supershift assays (data not shown) are indicated. C. Transfections in the indicated cell lines with luciferase reporter genes fused to the proximal TNF promoter (−200 to +87 nt) of the indicated primate species. Fold induction of luciferase activity in response to treatment with ionomycin (I) in 68-41 T cells, treatment with lipopolysaccharide (LPS) in J774 monocytic cells, or infection with *Mycobacterium tuberculosis* (MTb) in J774 monocytic cells is shown.

Fixed differences within or near the Sp1 site of the human TNF promoter (5′-CCCCGCCCCC-3′) at −172 to −163 bp relative to the start site of transcription are observed in representatives of the Asian apes (Pongidae and Hylobatidae) and NWM, but not in African great apes and OWM (Figure 7A). This site is one of two functional Sp1 binding sites that have been characterized in the human TNF promoter [3]. This particular Sp1 site is involved in regulation of the human TNF gene in cells of the monocyte/macrophage lineage in response to MTb infection and to treatment with LPS but has no apparent role in TNF gene regulation in T cells activated by calcium influx or by T cell receptor ligands [1]–[4]. We thus speculated that these differences may impact the regulation of these primate promoters in response to stimulation of monocytes by LPS and MTb.

To test this hypothesis, we first assayed the ability of these divergent Asian ape Sp1 sites to bind Sp1 proteins by performing electrophoretic mobility shift assays (EMSAs) with nuclear extracts from murine and human T cell lines and a monocytic murine cell line. For this analysis we used oligonucleotides matching the upstream Sp1 site of the two orangutan species and seven gibbon species that represented all four genera of gibbon as probes and compared their binding to that of an oligonucleotide matching the human upstream Sp1 site (Figure 7B). Using specific antibodies, we were able to examine the binding of Sp1 and Sp3 proteins, which are highly conserved and ubiquitously expressed in murine and human cell lines [71], to these sites. As shown in Figure 7B, the −168 G/A transition present in the Sp1 site in *Pongo pygmaeus, Pongo abelii*, and *Nomascus l. leucogenys* strongly interferes with the constitutive binding of Sp1 and Sp3 in T cells and monocytic cells as compared to the binding of these proteins to the human Sp1 site sequence. By contrast, the single −161 C/T transition found in *Hylobates lar, Hoolock leuconedys*, and *Symphalangus syndactylus* and the single −164 C/T transition found in *Hylobates moloch, Hylobates agilis*, and *Hylobates pileatus*, which are outside of the predicted core of the Sp1 site [3], does not inhibit binding of Sp1 and Sp3 to the oligonucleotides matching these sites (Figure 7B).

To examine if impaired binding of Sp1 and Sp3 to these sites impacted transcriptional regulation, we next prepared luciferase reporters matching sequences from −200 to +87 from *Pongo, Hylobates moloch*, and *Nomascus l. leucogenys*. We also prepared a TNF-luciferase reporter from *Gorilla*, which has an Sp1 site that is identical to the human sequence but which varies from the human proximal promoter sequence at position −9, and compared the activation of all of these reporters to an isogenic human TNF reporter construct [72]. Activation of the *Pongo* and *Nomascus* TNF promoters was impaired up to 60% relative to the human promoter in MTb-infected or LPS-treated monocytes but was not affected in T cells treated with ionomycin (Figure 7C). In all cases, transcriptional activation of the *Gorilla* TNF promoter was essentially identical to that of the human TNF promoter, indicating, in this context, that there is no functional role in TNF gene transcription for the −9 G/T fixed difference found in all non-human primates.

Thus, there is a strong correlation between transcription factor binding at the upstream Sp1 site and transcriptional activation in response to bacterial infection but not to calcium influx among these primate TNF promoters. This is consistent with our previous findings that Sp1 is recruited to the human TNF promoter and required for its activation in response to LPS or MTb stimulation in monocytic cells, but not for its activation in T cells in response to calcium influx [1]–[4].

## Discussion

The identification of fixed genetic differences with functional consequences, particularly those that underlie primate-specific and especially human-specific adaptive changes, is perhaps the most important goal of comparative primate genomics [44], [73]–[80]. Global analysis of gene transcription through the use of microarrays has revealed human-specific changes in gene expression relative to other primates [81]–[85]. Several studies in primates have also revealed human-specific changes within gene coding regions, which may underlie the evolution of specific human traits [45], [76]–[80]. For example, fixed human differences in the coding regions of the transcription factor FOXP2 and myosin heavy chain genes have been linked to the evolutionary development of speech in humans and the anatomy of the jaw, respectively [86], [87]. By contrast, humans have accumulated mutations that disrupt genes encoding olfactory receptors at rates roughly fourfold higher than in the great apes and other primates [88].

Although techniques like phylogenetic shadowing can be applied toprimate gene sequences to identify putative regulatory elements [9], [46], human-specific changes in non-coding regions have been more difficult to quantify than those in gene coding regions, since the effect of a given nucleotide change in a putative regulatory region cannot be readily deduced *a priori.* Despite the power of comparative genomics and microarray analysis, quantifying the effect of a given fixed genetic difference in a *cis*-regulatory region upon transcriptional regulation has remained elusive. Indeed, the transcriptional activity of human and chimpanzee promoters in transient transfection gene reporter assays does not always correlate with transcription profiles from microarray data [89].

Although sequence divergence between humans and other primates has been correlated with *cis*-regulatory regions known to bind transcription factors, these studies did not identify or quantify the binding of specific transcription factors [89]–[92]. In one study, primate SNPs (rather than fixed differences) in the CCR5 promoter were shown alter the affinity of the transcription factor NF-κB for its cognate DNA motif and have a modest effect upon basal levels of CCR5 transcription [30]. Here, we combined the strategies of comparative sequence analysis with biochemical assays to examine a well-characterized *cis*-regulatory region, the TNF promoter, in closely related primate species. By doing so, we were able not only to reveal how fixed genetic differences in the TNF promoter are markers of primate phylogeny, but also to isolate fixed genetic differences in the Asian apes–relative to humans and the African apes–that impact cell type- and stimulus-specific regulation of the TNF gene.

The fixed genetic differences that we identify in this study are particularly informative given the relative scarcity of divergent sequences among primate species and because of the importance of the gene in which they occur in the innate immune response. For example, a recent study of multiple intergenic autosomal regions in great apes and humans (16 to 22.4 kb) revealed no fixed differences between *Pongo pygmaeus* and *Pongo abelii* [93], even though Bornean and Sumatran orangutans are so divergent at the nuclear and mitochondrial genome level that they have been designated as distinct species [94]–[97].

Several TNF promoter SNPs are present in extended haplotypes that mark human ethnic populations and human ancestry [19]. For example, the −243 A/G SNP is part of a sub-Saharan African-derived extended haplotype, and the −856 C/T SNP is part of an Amerindian HLA haplotype [19]. Moreover, the −307 G/A SNP, which is a marker of an extended haplotype in European-derived populations [21] is not present in Amerindian populations, while it occurs in ∼10% of European-, Cambodian-, and African-derived populations [19]. In this context it is striking to consider that for the majority of SNPs in the human TNF promoter, the complementary (common or the rare) allele, occurs as a fixed difference in divergent primate clades.

A number of scenarios could explain why specific alleles of human TNF promoter SNPs are identical to fixed nucleotides at corresponding positions in primate TNF promoters. Moreover, different scenarios could apply to specific nucleotide positions. First, the variant SNP allele could represent the ancestral state by descent. The allele could have reached fixation in the human population due to genetic drift, or it might have appeared through recent positive selection. Second, the variant SNP allele could represent recent homoplasy, either through selection due to certain constraints on promoter sequence patterns or due to linkage with another genetic region, such as HLA.

Regardless of whether these variant human alleles are identical to divergent sequences in the primate lineage by descent or are identical by state, all of the nucleotide positions of these SNPs fall outside the core region of the TNF promoter known to be critical for TNF gene regulation. This observation is consistent with the accumulation of mutations under neutral evolution, which in turn may reflect linkage with other genes that have been positively selected [9], [20]. Indeed, since as described above, the majority of human TNF promoter SNPs have been found to be in linkage with HLA molecules [16], [17], [19], [21], SNPs may also be in linkage with other genes in the MHC locus with functional effects upon resistance and susceptibility to infectious disease. Linkage of TNF promoter SNPs with other genes is further supported by the fact that, despite the statistical association of human TNF promoter SNP alleles with clinical outcomes, attempts to determine a role for these SNPs in TNF gene transcription have produced inconsistent results, consistent with their location outside of the core promoter region. Even for −307 G/A, the most commonly examined SNP in the human TNF promoter, studies comparing the levels of TNF gene transcription (typically in response to LPS) induced by the two alleles have reached conflicting conclusions [24]–[26]. Linkage of SNPs at specific positions in the TNF promoter with other genes in the MHC locus may in turn underlie the fixation of specific nucleotides at these SNP positions and in other regions outside of the core TNF promoter during the evolution of the primate order.

Most notably, in addition to these fixed genetic differences outside of the proximal TNF promoter, we have identified fixed genetic differences in primate TNF promoters with direct impact upon TNF gene transcription. These occur within a region corresponding to the upstream Sp1 site (−173 to −163 relative to the start site of transcription) of the human TNF promoter (5′-CCCCGCCCCC-3′), which is completely conserved in the African apes (*Pan paniscus, Pan troglodytes*, and *Gorilla*). Fixed genetic differences are present in Asian apes (5′-CCCCACCCC-3′ in both *Pongo* species and 5′-CCCCACCTC-3′ in *Nomascus l. leucogenys*) that impair binding of Sp1 proteins to the site and the transcriptional activity of the TNF gene in a cell- and inducer- specific fashion. This recapitulates the regulation of the human TNF promoter [1]–[7]; binding of Sp1 protein to the upstream Sp1 site in the human TNF promoter, conserved in African ape TNF promoters, is required for induction of TNF transcription in response to LPS and MTb, but not for its expression in activated T cells [2]–[4].

Fixed genetic differences within or downstream of the upstream Sp1 site also occur in the gibbons, the other Asian apes examined in this study, although the exact nucleotide sequence varies even within the *Hylobates* genus (see Figures 1, 7A, and 7B). Variation at the upstream Sp1 site in the TNF promoter does not occur along geographic lines across all primate families, however: in all species of the Cercopithecidae (OWM) family examined in this study, including those native to Asia [Francois' langur (*Trachypithecus francoisi*) and the red-shanked douc (*Pygathrix nemaeus*)], the upstream Sp1 site is identical to the human sequence (see Figure 7A).

It is intriguing to speculate that the environmental conditions, perhaps consisting of novel pathogens that confronted the diverging populations derived from a common ancestor of Old World monkeys and the small and great apes, provided selective pressure on the TNF promoter locus. As subsequent evolution and speciation events proceeded, nucleotides involved in induction of TNF gene expression in response to bacterial infection (LPS or MTb) of monocytic cells, rather than activation of T cells, became fixed in the gibbon-orangutan and the African great ape clades.

For orangutans and gibbons, the considerable geographic flux of the Malay Archipelago region of southeast Asia over millions of years, alternately isolating and reuniting local animal populations in the course of their adaptation and speciation [96], may have contributed to fixed genetic differences at the upstream Sp1 site of the TNF promoter. Indeed, based on comparisons between human and chimpanzee genome sequences, positive selection has been proposed to have a greater influence in 5′ untranslated regions as compared to protein coding regions [98]. Furthermore, co-evolution between hosts and pathogens has been proposed for primates and certain viruses and parasites [99], [100]; such reciprocal changes within a specific disease environment may have also influenced geographically dependent fixed differences in the primate TNF promoter. Finally, in addition to their direct functional effect upon TNF transcription, the Asian ape- and African ape-specific TNF promoter alleles may reflect linkage disequilibrium with neighboring genes that were under selective evolutionary pressure.

As genomic sequence data is acquired for different primate species, including data for extinct species such as *Homo neanderthalensis* [101], [102], we expect that other functional genetic changes marking points of divergence and speciation among the primate lineages will be discovered, contributing new insights into the genetic basis of the development of human and primate phenotypes. It will also be of interest to integrate information from primate comparative genomics with data on genetic differences among humans as these accumulate [103], [104]. Furthermore, given that multiple primate species in this study are endangered–*Gorilla beringei beringei, Gorilla beringei graueri,* and *Pongo abelii* are among the 25 most endangered primate species in the world [105]–our results underscore the importance of primate conservation in understanding the evolution and development of key genes in the human immune response.

## Materials and Methods

### Primate samples

Cell lines and samples of blood or DNA from representative individuals were obtained for the primate species and subspecies as described in Figure 2. To confirm the subspecies of chimpanzees, we amplified 498 bp of the mitochondrial D-loop region (control region) corresponding to the positions 15,998–16,497. PCR products were sequenced and these sequences were analyzed together with a representative of each of the subspecies reported in GenBank (*Pan troglodytes troglodytes*, AF102683; *P. t. schweinfurthii*, AF102687; *P. t. verus*, AF315499; *P. t. vellerosus*, AF315498) on a phylogenetic tree using maximum likelihood methods with PHYLIP 3.65 package [106]. Samples associating in a clear cluster with a reference subspecies sequence were assigned to that subspecies, as described previously [107]. DNA from orangutans was obtained from cell lines described previously [93], [95].

### Sequencing of primate TNF promoters

Genomic DNA was isolated from whole blood using the QIAamp DNA Blood kit (Qiagen). The TNF promoter was amplified using the following primer sets: for human, apes, and OWM, TNF-F1 (5′-AGT GAG AAC TTC CCA GTC TAT CTA AG-3′) and TNF-R1 (5′-CCG TGG GTC AGT ATG TGA GA-3′) [19]; for NWM and lemur, TNF-F2 (5′-CCC AAT AAA CCT CTT TTC TCT GA-3′), TNF-F3 (5′-TTG GAA GCC AAG ACT GAA ACC-3′) and TNF-R2 (5′-GTG CCA ACA ACT GCC TTT A-3′). In the case of samples for which just one individual was available, the PCR product was subcloned into the p-GEM-T easy vector (Promega) and at least 3 colonies were analyzed. Sequences were obtained from both directions from −1153 to +69 using a Perkin-Elmer 377 automatic sequencer.

### Plasmids

Using PCR, we amplified primate TNF promoters from −200 to +87 nt relative to the transcription start site and the NheI-BglII restriction fragment was subcloned into pGL3 Basic (Promega) and sequenced. Constructs for transient transfection were thus isogenic to the human −200 TNF-Luc reporter [72]. Plasmids were isolated with EndoFree plasmid kits (Qiagen) for use in the J774A.1 cell line and Maxiprep plasmid kits (Promega) for Jurkat and 68-41 cell lines.

### Cell culture and Transfection

The Jurkat T cell line (ATCC) and T cell hybridoma 68-41(a gift from Masato Kubo, Science University of Tokyo, Tokyo, Japan) were maintained at 37°C, 5% CO_2_, in RPMI 1640 (Gibco) supplemented with penicillin, streptomycin, 10% fetal bovine serum, and 2mM L-glutamine. Transfections were performed using DEAE-Dextran as described [5]. After 6 hours, cells were stimulated with 1 µM Ionomycin (Calbiochem) for approximately 16 hours. The J774A.1 Monocyte cell line (ATCC) was maintained at 37°C, 5% CO_2_ in Dulbecco's MEM media (Gibco) suplemented with penicillin, streptomycin, 10% fetal bovine serum. Transfections were performed using FuGene6 (Roche) using a 3∶2 ratio (pGL3: pR-TK Renilla) according to the manufacturer's protocol. Eight hours after transfection, cells were treated with 2 µg/ml of LPS (0111:B4, Sigma) or 5 µg/ml of H37Rv whole sonicate of *M. tuberculosis* (a gift from Chris Dascher at Brigham and Women's Hospital, Boston MA, USA) and harvested 16 hours later. Luciferase assays were performed according to the manufacturer's instructions (Dual Luciferase Reporter Assay System; Promega) using a Dynex luminometer, with Renilla luciferace (pR-TK) as an internal control.

### Electrophoretic Mobility Shift Assay

Nuclear extracts were prepared from Jurkat and 68-41 cell lines after stimulation for 2 hours with ionomycin, and from J774A.1 cells after stimulation for 2 hours with LPS or *M. tuberculosis* as described above. Nuclear extracts were prepared, and electrophoretic mobility shift assays were performed, as previously described [5]. Sp1 sense and anti-sense oligonucleotide probe sequences were as follows: human, 5′-AAA TCC CCG CCC CCG CGA TGG-3′ and 5′-CCA TCG CGG GGG CGG GGA TTT-3′; *Gorilla*, 5′-AAA TCC CCG CCC CCG CGA TGG-3′ and 5′-CCA TCG CGG GGG CGG GGA TTT-3′; *Pongo*, 5′-AAA TCC CCA CCC CCG TGA TGG-3′ and 5′-CCA TCA CGG GGG TGG GGA TTT-3′; *Hylobates moloch*, 5′-AAA TCC CCG CCC CCG TGA TGG-3′ and 5′-CCA TCA CGG GGG CGG GGA TTT-3′; *Hoolock leuconedys*, 5′-AAA TCC CCG CCC TCG CGA TGG-3′ and 5′-CCA TCG CGA GGG CGG GGA TTT-3′; *Nomascus l. leucogenys*, 5′-AAA TCC CCA CCC TCG CGA TGG-3′ and 5′-CCA TCG CGA GGG TGG GGA TTT-3′.

### Phylogenetic and Statistical analysis

The sequences were aligned with the CLUSTALW program, version 1.7 [108]. Phylogenetic analyses were performed using the PHYLIP v. 3.57c program [106]. First, a matrix of distances were obtained using the Kimura 2-parameter model, and then we generated 1000 replications by bootstrap analysis. Phylogenetic trees were generated using Neighbor-joining, and a consensus tree was finally obtained. We generated a second set of bootstrap values using heuristic parsimony with the PAUP v.3.1 program [109]. Phylogenetic shadowing of the TNF promoter was performed using the eShadow program [47]. The MSA plot was generated using TNF promoter sequences −1153 to +69 from human and all other primate species examined in the studies with a sliding window of 50 nt. Maximum variation was 10%, minimal length 80 nt.

## Supporting Information

Figure S1 Complete alignment of the TNF promoter sequences from primate species used in this study. Numbering corresponds to the human TNF promoter sequence, −1153 to +69 nt relative to the start site of transcription, shown at the top. Species and subspecies are indicated, along with nucleotide changes, conserved positions (.), and deletions (−). All alterations from the human sequence represent fixed differences, except positions in which SNPs were detected in 40% or more of the individuals examined, which are denoted R (A or G), Y (C or T), M (A or C), or S (C or G). The −1027 to −1029 deletion found as a SNP in *P. t. troglodytes* is also shown. On the human TNF promoter sequence, positions of human SNPs and every hundredth base pair position are in boldface, and every tenth base pair position is underlined.
(0.15 MB DOC)

Table S1 Total fixed differences among the primate TNF promoters. Positions of single nucleotide changes and discrete insertions or deletions, relative to the consensus human TNF promoter sequence, observed in this study are shown.
(0.04 MB PDF)

Table S2 SNPs in primate TNF promoters. For each of the indicated species or subspecies, the position, sequence change, and frequency (in the total number of individuals examined) of each SNP detected in the TNF promoter is shown. SNPs in which the wild type allele corresponds to the sequence found in the other subspecies of a given species are shown, while the mutant alleles that correspond to the human sequence are noted with an asterisk.
(0.07 MB PDF)

Table S3 Fixed differences in the TNF promoter that mark primate clades. Total fixed genetic differences in the TNF promoter that are unique to and that are completely conserved within the indicated primate taxa are shown. A. For each position, the sequence found within and outside of the indicated clades is shown. B. For each position, the sequence found in humans and in the indicated genus or clade is shown. The Cebidae family includes *Cebus capucinus* and *Saimiri sciureus*; the Atelinae subfamily, *Ateles geoffroyi* and *Lagothrix lagotricha*. See Figure 6 for diagram of evolutionary context.
(0.03 MB PDF)
