# Supplementary material for: Primate TNF Promoters Reveal Markers of Phylogeny and Evolution of Innate Immunity
Source: PLoS One. 2007 Jul 18;2(7):e621. doi: 10.1371/journal.pone.0000621 (PMC1905939; doi:10.1371/journal.pone.0000621)
Supplement: Figure S1 — Complete alignment of the TNF promoter sequences from primate species used in this study. Numbering corresponds to the human TNF promoter sequence, −1153 to +69 nt relative to the start site of transcription, shown at the top. Species and subspecies are indicated, along with nucleotide changes, conserved positions (.), and deletions (−). All alterations from the human sequence represent fixed differences, except positions in which SNPs were detected in 40% or more of the individuals examined, which are denoted R (A or G), Y (C or T), M (A or C), or S (C or G). The −1027 to −1029 deletion found as a SNP in P. t. troglodytes is also shown. On the human TNF promoter sequence, positions of human SNPs and every hundredth base pair position are in boldface, and every tenth base pair position is underlined. (0.15 MB DOC) [file pone.0000621.s001.doc]

-1153 -1100 -1072 -1045

*Homo sapiens* GGGAGCAAGAGCTGTGGGGAGAACAAAAGGATAA-GGGCTCAGAGAGCTTCAGGGATATGTGATGGACTCACCAGGTGAGGC**C**GCCAGACTGCTGCAGGGGAAGCAAAGG

*Pan paniscus* ..................................-...........................................................................

*Pan troglodytes verus* ..................................-...........................................................................

*Pan t. troglodytes* ..................................-...........................................................................

*Pan t. vellerosus* ..................................-...........................................................................

*Pan t. schweinfurthii* ..................................-...........................................................................

*Gorilla gorilla gorilla* ..................................-...........................................................................

*Gorilla beringei graueri*..................................-...........................................................................

*Gorilla b. beringei* ..................................-.......**T**...................................................................

*Pongo pygmaeus* ..................................**A**.....................................**G**.....................................

*Pongo abelii*  ..................................**A**.....................................**G**.....................................

*Hylobates lar* ..................................**A**...............................................**T**.......**G**...................

*Hylobates moloch* ..................................**A**............**T**..................................**T**.......**G**...................

*Hylobates agilis*  ..................................**A**............**T**..................................**T**.......**G**...................

*Hylobates pileatus* ..................................**A**............**T**..................................**T**.......**G**...................

*Hoolock leuconedys* ..................................**A**............**T**..................................**T**.....**G**.**G**...................

*Nomascus l. leucogenys* ..................................**A**............**T**..................................**T**.......**G**...................

*Symphalangus syndactylus*..................................**A**............**T**..................................**T**.......**G**...................

*Chlorocebus sabaeus* ..............................**G**...**A**.....................**C**.....................................................

*Chlorocebus tantalus* ..................................**A**.....................**C**.....................................................

*Chlorocebus pygerythrus* ..................................**A**.....................**C**............................**T**........................

*Cercopithecus diana* ..................................**A**.....................**C**.....................................................

*Cercocebus torquatus* ..................................**G**.....................**C**.....................................................

*Papio hamadrayas* ..................................**G**........**G**............**C**.....................................................

*Macaca mulatta* ..................................**G**.....................**C**.....................................................

*Ateles geoffroyi* ........**C**.........**C**.....**G**.**GG**......**A**...............................**G**...............**A**.....**G**.....................

*Lagothrix lagotricha* ........**C**.........**C**.....**G**.**GG**......**AT**........................**C**.....**G**...............**A**.....**G**.....................

*Cebus capucinus* .....**G**..**C**.......**A**.**C**.......**G**.......**A**...............................**G**...**G**...........**A**.....**G**.....................

*Saimiri sciureus* .....**G**..**C**.......**A**.**C**.......**G**.......**A**...............................**G**...**G**...........**A**.....**G**.....................

*Aotus trivirgatus* ........**C**.........**C**.......**G**.......**AA**................**A**..........**C**..**G**...............**A**...........................

***** ** ******* * ***** * ** *** ****** *** **** *** *** ** ** *** * ********* ** ** * *******************

-1044 -1030 -1000 -937

*Homo sapiens* AGAAGCTGAGAAGATGAAGGAAAAGTCAGGGTCTGGAGGGGCGG**G**GGTCAGGGAGCTCCTGGGA--GATATGGCCACATGTAGCGGCTCTGAGGAATGGGTTACAGGAGA

*Pan paniscus* ..............**A**.................................................--............................................

*Pan troglodytes verus* ..............**A**.................................................--............................................

*Pan t. troglodytes* ..............**A**---..............................................--............................................

*Pan t. vellerosus*  ..............**A**.................................................--............................................

*Pan t. schweinfurthii*  ..............**A**.................................................--............................................

*Gorilla gorilla gorilla* ..............**C**.................................................--..................**A**..................**A**......

*Gorilla beringei graueri*..............**C**.................................................--..................**A**..................**A**......

*Gorilla b. beringei* ..............**C**.................................................--..................**A**..................**A**......

*Pongo pygmaeus* ..............**CA**................................................--.................**T**..........................

*Pongo abelii*  ..............**CA**................................................--.................**T**..........................

*Hylobates lar*  ..............**CA**.........................**A**......................--.................**T**..........................

*Hylobates moloch*  ..............**CA**.........................**A**......................--.................**T**..........................

*Hylobates agilis*  ..............**CA**.........................**A**......................--.................**T**..........................

*Hylobates pileatus*  ..............**CA**.........................**A**......................--.................**T**..........................

*Hoolock leuconedys*  ..............**CA**................................................--.................**T**..........................

*Nomascus l. leucogenys* ..............**CA**................................................--.................**T**..........................

*Symphalangus syndactylus*..............**CA**................................................--.................**T**..........................

*Chlorocebus sabaeus* ..............**CA**........**A**..........**A**............................--............**A**....**T**........................**C**.

*Chlorocebus tantalus* ..............**CA**...................**A**............................--............**A**....**T**..........................

*Chlorocebus pygerythrus* ..............**CA**...................**A**............................--............**A**....**T**..........................

*Cercopithecus diana* .........**T**....**CA**...................**A**............................--............**A**....**T**..........................

*Cercocebus torquatus* ..............**CA**...................**A**............................--............**A**....**T**..........................

*Papio hamadrayas*  .........**T**....**CA**...................**A**............................--............**A**....**T**..........................

*Macaca mulatta*  .**T**............**CA**...................**A**............................--............**A**....**T**..........................

*Ateles geoffroyi*  ..**G**...........**CA**........................................**CG**......--.**G**.....**T**.........**T**.................**G**........

*Lagothrix lagotricha*  ..**G**...........**CA**........................................**C**.......--.......**T**.........**T**.................**G**........

*Cebus capucinus*  ..**G**..........**GCA**...................**C**....................**CG**.**C**...**G**--.......**T**.........**T**.................**G**........

*Saimiri sciureus*  ..**G**..........**GCA**...................**C**....................**CG**.**C**...**GGG**.......**T**.........**T**.................**G**........

*Aotus trivirgatus*  ..............**CA**.------.......**A**...........**A**.............**CG**......--....**C**..**T**.........**T**.................**G**........

* ****** *** * ***** **** ***** ************* * *** * ** ** **** **** **************** * **** *

-936 -900 -862 -856 -827

*Homo sapiens* CCTCTGGGGAGATGTGACCACAGCAATGGGTAGGAG**A**ATGTCCAGGGCTATGGAAGTCGAGTATGGGGACCCCC**C**CTTAA**C**GAAGACAGGGCCATGTAGAGGGCCCCAGG

*Pan paniscus* ................................................................................**Y**.............................

*Pan troglodytes verus*  ..............................................................................................................

*Pan t. troglodytes*  ..............................................................................................................

*Pan t. vellerosus*  ..............................................................................................................

*Pan t. schweinfurthii*  ..............................................................................................................

*Gorilla gorilla gorilla* ........................................................................**T**.....................................

*Gorilla beringei graueri*........................................................................**T**.....................................

*Gorilla b. beringei* ........................................................................**T**.....................................

*Pongo pygmaeus* ..........................**A**...........................**G**.....................--..........**A**.....................

*Pongo abelii*  ..........................**A**...........................**G**..**T**.................---..........**A**.....................

*Hylobates lar*  ..........................**A**................**G**..........**G**.................**T**...**CC**................................

*Hylobates moloch*  ..........................**A**................**G**..........**G**.................**T**...**CC**................................

*Hylobates agilis*  ..........................**A**................**G**..........**G**.................**T**...**CC**................................

*Hylobates pileatus*  ..........................**A**...............**TG**..........**G**.................**T**...**CC**................................

*Hoolock leuconedys*  ..........................**A**...........................**G**................**TT**...**CC**................................

*Nomascus l. leucogenys* ..........................**A**...........................**G**.................**T**...**CC**................................

*Symphalangus syndactylus*..........................**A**...........................**G**.................**T**...**CC**...**A**............................

*Chlorocebus sabaeus* ..........................**A**...........**A**..............**GG**..**T**..................---**G**.**C**........................**T**...

*Chlorocebus tantalus* ..........................**A**...........**A**..............**GG**..**T**..................---**G**.**C**........................**T**...

*Chlorocebus pygerythrus* .................**M**........**A**...........**A**..............**GG**..**T**..................---**G**.**C**....**S**...................**T**...

*Cercopithecus diana*  ..........................**A**..........................**GG**..**T**..................---**G**.**C**........................**T**...

*Cercocebus torquatus* ..........................**A**......................**G**...**GG**..**T**.................**T**---**G**.**C**........................**T**...

*Papio hamadrayas*  ..........................**AA**.........................**GG**..**T**.................**TG**---.**C**........................**T**...

*Macaca mulatta*  ..........................**A**..........................**GG**..**T**...................--**G**.**C**........................**T**...

*Ateles geoffroyi*  ........**A**....**A**............**A**..................**A**.......**GG**.......**G**...........**G**..---.**C**.............**AC**.............

*Lagothrix lagotricha*  ........**A**.................**A**..................**A**.......**GG**.......**G**...........**G**..---.**C**.............**AC**.............

*Cebus capucinus*  ..........................**A**..........................**GG**.......**G**....**T**......**A**..---.**C**.............**AC**............**A**

*Saimiri sciureus*  ..........................**A**..........................**GG**.......**G**....**T**......**A**..---.**C**.............**AC**............**A**

*Aotus trivirgatus*  ....................**TG**....**A**...................**A**......**GG**.......**G**..........**TG**..---.**C**........**A**....**AC**.............

******** **** *** ** **** ********** *** * ** *** ** **** **** *** **** * * **** ********* **

-826 -800 -720

*Homo sapiens* GAGTGAAAGAGCCTCCAGGACCTCCA**G**GTATGGAAT---ACAGGGGACGTTTAAGAAGATATGGCCACACACTGGGGCCCTGAGAAGTGAGAGCTTCATGAAAAAAATCA

*Pan paniscus*  ....................................---.......................................................................

*Pan troglodytes verus*  ....................................---.......................................................................

*Pan t. troglodytes*  ...........................**.**........---.......................................................................

*Pan t. vellerosus*  ...........................**.**........---.......................................................................

*Pan t. schweinfurthii*  ....................................---............**Y**..........................................................

*Gorilla gorilla gorilla* ....................................---....................**C**.............................................**T**....

*Gorilla beringei graueri*....................................---....................**C**.............................................**T**....

*Gorilla b. beringei* ....................................---..................................................................**T**....

*Pongo pygmaeus* ....................................---...**C**.........**C**.........................................................

*Pongo abelii*  ....................................---...**C**.........**C**.........................................................

*Hylobates lar*  ....................................---...................**C**...................................................

*Hylobates moloch*  ....................................---.......................................................................

*Hylobates agilis*  ....................................---.......................................................................

*Hylobates pileatus*  ....................................---...................**C**...................................................

*Hoolock leuconedys*  ....................................---.......................................................................

*Nomascus l. leucogenys* ....................................---.......................................................................

*Symphalangus syndactylus*....................................---.......................................................................

*Chlorocebus sabaeus* .................**A**......**T**...........---.........**C**......................**T**.....................**T**................

*Chlorocebus tantalus* .................**A**......**T**...........---................................**T**.**.**...................**T**................

*Chlorocebus pygerythrus* .................**A**......**T**...........---................................**T**.**R**...................**T**................

*Cercopithecus diana* ........................**T**...........---................................**T**.....................**T**................

*Cercocebus torquatus* ........................**T**...........---................................**T**.....................**T**................

*Papio hamadrayas*  ........................**T**...........---................................**T**.....................**T**................

*Macaca mulatta*  ...**C**....................**T**...........---................................**T**.....................**T**................

*Ateles geoffroyi*  ..............................**C**.....---........**TA**..........**C**...................**A**......**A**...............**C**...-...

*Lagothrix lagotricha*  ...........**T**........................---........**T**...........**C**...................**G**......**A**...............**C**...-...

*Cebus capucinus*  ....................................**AAT**........**T**...........**C**...........**T**......**TG**......**A**...........**CA**..**C**...-...

*Saimiri sciureus*  ....................................---........**T**...........**C**........**G**..**T**......**TG**......**A**...........**CA**..**C**...-...

*Aotus trivirgatus*  ...............................**C**....---........**T**...........**C**........**G**......**A**..**GG**......**A**...............**C**...-...

*** ******* ***** ****** ** ** **** *** **** ** ***** ******** ** * * ** ****** ****** **** ** ** ***

-719 -700 -610

*Homo sapiens* GGGACCCCAGAGTTCCTTG**G**AAGCCAAGACTGAAACCAGCATTATGAGTCTCCGGGTCAGAATGAAAGAAGAAGGCCTGCCCCAGTGGGGTCTGTGAATTCCCGGGGGTG

*Pan paniscus*  ..............................................................................................................

*Pan troglodytes verus*  ....................................................**T**.........................................................

*Pan t. troglodytes*  ....................................................**T**.........................................................

*Pan t. vellerosus*  ....................................................**T**.........................................................

*Pan t. schweinfurthii*  ....................................................**T**.........................................................

*Gorilla gorilla gorilla* ....................................................**T**.........................................................

*Gorilla beringei graueri*....................................................**T**.........................................................

*Gorilla b. beringei* ....................................................**T**.........................................................

*Pongo pygmaeus* ...........................................**G**...........................................................**A**......

*Pongo abelii*  ...........................................**G**...........................................................**A**......

*Hylobates lar*  ...........................................**G**......................................**T**....................**A**......

*Hylobates moloch*  ...........................................**G**......................................**T**....................**A**......

*Hylobates agilis*  ...........................................**G**......................................**T**....................**A**......

*Hylobates pileatus*  ...........................................**G**......................................**T**...........................

*Hoolock leuconedys*  ...........................................**G**......................................**T**....................**A**......

*Nomascus l. leucogenys* ...........................................**G**......................................**T**....................**A**......

*Symphalangus syndactylus*...........................................**G**......................................**T**....................**A**......

*Chlorocebus sabaeus* .........**C**.................................**G**......**C**..**C**.......**G**................................................

*Chlorocebus tantalus* .........**A**.................................**G**......**C**..**C**.....**A**.**G**.....**A**..**A**.**G**................................**T**....

*Chlorocebus pygerythrus* ...........................................**G**......**C**..........**G**.........**TT**..............................**A.T**....

*Cercopithecus diana* ..............**G**............................**G**......**C**..........**G**.................**T**.........................**T**....

*Cercocebus torquatus* ..........**C**................................**G**......**C**........**T**.**C**...........................................**T**....

*Papio hamadrayas*  ...........................................**G**......**C**..........**G**...........................................**T**....

*Macaca mulatta*  ...........................................**G**......**C**..........**G**...........................................**T**....

*Ateles geoffroyi*  ..............**T**..........................**G**.**G**......**C**.**A**....**A**..........**G**.....**C**.................**C**..............**C**..

*Lagothrix lagotricha*  ..............**T**..........................**G**.**G**......**C**.**A**...............**G**.....**C**......**T**..........**C**..............**C**..

*Cebus capucinus*  ..............**T**..........................**G**.**G**......**C**.**A**...............**G**.....**C**...**AT**..-.........**C**...........**A**..**C**..

*Saimiri sciureus*  ..............**T**..........................**G**.**G**......**C**.**A**...............**G**.....**C**...**AT**..-.........**C**..............**C**..

*Aotus trivirgatus*  ..............**T**..........................**G**.**G**......**C**.**G**...............**G**.....-----............................**C**..

********* *** ************************** * ****** * *** * * ***** * * * ********* ********** * **

-609 -600 -574 -516

*Homo sapiens* ATTTCACTC**C**CCGGGGCTGTCCCAGGCTTGTCCCT**G**CTACCCCCACCCAGCCTTTCCTG-AGGCCTCAAGCCTGCCACCAAGC---------------CCCCAGCTCCTT

*Pan paniscus*  ...........................................................-......................C---------------............

*Pan troglodytes verus*  ...........................................................-......................C---------------............

*Pan t. troglodytes*  ...........................................................-......................C---------------............

*Pan t. vellerosus*  ...........................................................-......................C---------------............

*Pan t. schweinfurthii*  ...........................................................-......................C---------------............

*Gorilla gorilla gorilla* ...........................................................-......................C---------------.........**T**..

*Gorilla beringei graueri*...........................................................-......................C---------------............

*Gorilla b. beringei* ...........................................................-......................C---------------......**T**..**T**..

*Pongo pygmaeus* ......................................................**C**....-......**TG**..............C---------------.....**A**......

*Pongo abelii*  ......................................................**C**....-......**TG**..............C---------------.....**A**......

*Hylobates lar*  ......................................................**C**....**T**.......**G**..............C---------------............

*Hylobates moloch*  ......................................................**C**....-.......**G**..............C---------------............

*Hylobates agilis*  ......................................................**C**....-.......**G**..............C---------------............

*Hylobates pileatus*  ..........................................**T**...........**C**....-.......**G**..............C---------------............

*Hoolock leuconedys*  ......................................................**C**....-.......**G**..............C---------------............

*Nomascus l. leucogenys*  ......................................................**C**....-.......**G**..............C---------------............

*Symphalangus syndactylus*......................................................**C**....-.......**G**..............C---------------............

*Chlorocebus sabaeus* ......................................................**C**....-.....**CTG**........**C**.....C---------------............

*Chlorocebus tantalus* ......................................................**C**....-.....**CTG**........**C**.....C---------------............

*Chlorocebus pygerythrus* ..........................................**T**...........**C**....-.....**CTG**........**C**.....G---------------.........**T**..

*Cercopithecus diana* ......................................................**C**....-.....**CTG**.**A**......**C**.....C---------------............

*Cercocebus torquatus* ............**A**.........................................**C**....-.....**GTG**...**T**....**C**.....C---------------.........**T**..

*Papio hamadrayas*  ......................................................**C**....-.....**CTG**........**C**.....C---------------............

*Macaca mulatta*  .............................................**T**........**C**....-.....**CTG**........**C**.....C---------------............

*Ateles geoffroyi*  ...........**T**................**C**........................**AC**..**C**.-...**T**.**CTG**........**C**.....**TCTGGAGCTGCCCTGT**...**TG**.......

*Lagothrix lagotricha*  ...........**T**................**C**..............**T**......**T**..**AC**..**C**.-...**T**.**CTG**........**C**.....**TCTGGAGCTGCCCTGT**...**TG**.......

*Cebus capucinus*  ...........**T**.........................................**AC**..**C**.-...**T**.**CTG**..**T**...**T**.**C**...**C**.**TATGGAGCTGCCCTGT**...**TG**.......

*Saimiri sciureus*  ...........**T**.........................................**AC**....-...**T**.**CTG**........**C**.....**TATGGAGCTGCCCTGT**...**TG**.......

*Aotus trivirgatus*  ...........**T**.........................................**AC**..**C**.-...**T**.**CTG**.**CG**.....**C**...**G**.**TATGGAGCTGCCCTGT**...**TG**.......

*********** *************** ************* * **** ** ** * *** * * ** * *** * *** ** **

-515 -500 -408

*Homo sapiens* CTCCCCGCAGGGACC**C**AAACACAGGCCTCAGGACTCAACACAGCTTTT--CCCTCCAACCCCGTTTTCTCTCCCTCAAGGACTCAGCTTTCTGAAGCCCCTCCCAGTTCT

*Pan paniscus*  ................................................--............................................................

*Pan troglodytes verus*  ................................................--............................................................

*Pan t. troglodytes*  ...........................................**.**....--............................................................

*Pan t. vellerosus*  ................................................--............................................................

*Pan t. schweinfurthii*  ................................................--............................................................

*Gorilla gorilla gorilla* ................................................--............................................................

*Gorilla beringei graueri*................................................--............................................................

*Gorilla b. beringei* ................................................--.......................**A**....................................

*Pongo pygmaeus* ......**C**........**A**................................--............................................................

*Pongo abelii*  ......**C**........**A**................................--............................................................

*Hylobates lar*  ......**C**....................................**A**....--....................................**G**..................**T**....

*Hylobates moloch*  ......**C**.........................................--....................................**G**..................**T**....

*Hylobates agilis*  ......**C**.........................................--....................................**G**..................**T**....

*Hylobates pileatus*  ......**C**.........................................--...........**T**...........**A**............**G**..................**T**....

*Hoolock leuconedys*  ......**C**.........................................--....................................**G**.......................

*Nomascus l. leucogenys*  ......**C**.........................................--....................................**G**..................**T**....

*Symphalangus syndactylus*..**T**...**C**...............................**A**.........--....................................**G**.......................

*Chlorocebus sabaeus* ......**CA**...**A**....................................**CT**............**A**........**A**......................................

*Chlorocebus tantalus* ......**CA**...**A**....................................**CT**............**A**........**A**......................................

*Chlorocebus pygerythrus* **T**.....**CA**...**A**....................................**CT**............**A**........**A**......................................

*Cercopithecus diana* ......**CA**...**A**....................................**CT**............**A**........**A**......................................

*Cercocebus torquatus* ......**CA**...**A**........**G**...........................**CT**............**A**........**A**......................................

*Papio hamadrayas*  ......**CA**...**A**........**G**..**A**........................**CT**............**A**........**A**......................................

*Macaca mulatta*  ......**CA**...**A**........**G**...........................**CT**............**A**........**AT**.....................................

*Ateles geoffroyi*  ....**T**.**C**.........................................--.........**T**..**A**............**TG**.........**T**.......................

*Lagothrix lagotricha*  ....**T**.**C**.........................................--.........**T**..**A**............**TG**.........**T**............**T**..........

*Cebus capucinus*  ....**T**.**C**.................**A**................**G**......--.........**T**.........**G**.....**TG**.................................

*Saimiri sciureus*  ....**T**.**C**.........................................--.....**T**...**T**..**A**...........**CTG**.................................

*Aotus trivirgatus*  ...**TTTC**......................................**A**..--.........**T**..**A**............**TG**.................................

* *** *** **** ** ************* ** * * ** ***** *** * ****** * ********* ************ ***** ****

-407 -400 -375 -307 -299

*Homo sapiens* AGTTCTA**T**CTTTTTCCTGCATCCTGTCTGGAA**G**TTAGAAGGAAACAGACCACAGACCTGGTCCCCAAAAGAAATGGAGGCAATAGGTTTTGAGGGGCATG**G**GGACGGGG-

*Pan paniscus*  .............................................................................................................-

*Pan troglodytes verus*  .............................................................................................................-

*Pan t. troglodytes*  ................................................................................**G**............................-

*Pan t. vellerosus*  .............................................................................................................-

*Pan t. schweinfurthii*  .............................................................................................................-

*Gorilla gorilla gorilla* .............................................................................................................-

*Gorilla beringei graueri*.............................................................................................................-

*Gorilla b. beringei* .............................................................................................................-

*Pongo pygmaeus* .......**C**.................................................................**CA**.................................**A**-

*Pongo abelii*  .......**C**.................................................................**CA**.................................**A**-

*Hylobates lar*  .........................................................................**C**...................................-

*Hylobates moloch*  ...................................................**T**.....................**C**...................................-

*Hylobates agilis*  ...................................................**T**.....................**C**...................................-

*Hylobates pileatus*  ...................................................**T**.....................**C**...................................-

*Hoolock leuconedys*  ...................................................**T**.....................**C**...................................-

*Nomascus l. leucogenys*  ............**C**......................................**T**.....................**C**...................................-

*Symphalangus syndactylus*...................................................**T**.....................**C**...................................-

*Chlorocebus sabaeus* .............................**A**..**A**.**C**...**G**...........................**G**..................**RC**...........**G**.**A**.**C**.....**A**-

*Chlorocebus tantalus* .............................**A**..**A**.**C**...**G**................**Y**..........**G**...................**C**...........**G**.**A**.**C**.....**A**-

*Chlorocebus pygerythrus* .............................**A**..**A**.**C**...**G**................**.**..........**G**...................**C**...........**G**.**A**.**C**.**.**...**A**-

*Cercopithecus diana* .............................**A**..**A**.**C**...**G**...........................**G**...................**C**...........**G**.**A**.......**A**-

*Cercocebus torquatus* ................................**A**.**C**...**G**...............................................**C**...........**G**.**A**.......**A**-

*Papio hamadrayas*  ................................**A**.**C**...**G**...........................**G**...................**C**...........**G**.**A**.......**A**-

*Macaca mulatta*  ................................**A**.**C**...**G**...........................**G**.....**C**.............**C**...........**G**.**A**.......**A**-

*Ateles geoffroyi*  **CC**..........................**A**..**CA**.**CG**..**G**...................................**TC**..**A**.......**C**................**GAA**...**A**

*Lagothrix lagotricha*  **CC**..........................**A**..**CA**.**CG**..**G**...................................**TC**..**A**...**C**...**C**................**GAA**...**A**

*Cebus capucinus*  **CCC**..----.....................**GCA**.**C**...**G**...................................**TC**..**A**.......**C**................**GAAA**..**A**

*Saimiri sciureus*  **CCC**..----......................**CA**.**C**...**G**...................................**TC**..**A**.......**C**................**GAA**...**A**

*Aotus trivirgatus*  **CCC**............................**CA**.**CG**..**G**...................................**TC**..**A**...**C**...**C**................**GAC**----

** *** *************** * ** ************ *** ********** ***** ** * * ** *********** * *

-298 -243 -237 -200 -190

*Homo sapiens* -TTCAGCCTCCAGGGTCCTACACACAAATCAGTCAGTGGCCCAGAAGACCCCCCTC**G**GAATC**G**GAGCAGGGAGGATGGGGAGTGTGAGGGGTATCCTTG**A**TGCTTGTGTG

*Pan paniscus*  -.............................................................................................................

*Pan troglodytes verus*  -.............................................................................................................

*Pan t. troglodytes* -.............................................................................................................

*Pan t. vellerosus*  -.............................................................................................................

*Pan t. schweinfurthii*  -.............................................................................................................

*Gorilla gorilla gorilla* -.............................................................................................................

*Gorilla beringei graueri-.*............................................................................................................

*Gorilla b. beringei* -.............................................................................................................

*Pongo pygmaeus* -.....................**T**.**T**..........**C**......................................**G**..............................**A**....

*Pongo abelii*  -.....................**T**.**T**..........**C**......................................**G**..............................**A**....

*Hylobates lar*  -.........**T**................**G**.......**C**.............................**A**........**G**...................................

*Hylobates moloch*  -.........**T**........................**C**..............**T**.......................**G**......**C**............................

*Hylobates agilis*  -.........**T**........................**C**..............**T**.......................**G**......**C**............................

*Hylobates pileatus*  -.........**T**........................**C**...**A**..................................**G**......**C**............................

*Hoolock leuconedys*  -.........**T**........................**C.**.....................................**G**...**C**...............................

*Nomascus l. leucogenys*  -.........**T**........................**C**......................................**G**...................................

*Symphalangus syndactylus*-.........**T**........................**C**..........................**A**..**A**........**G**...................................

*Chlorocebus sabaeus* -....**A**.......**A**....**CG**................................**T**...................**T**.**G**.......**A**......**A**................**G**...

*Chlorocebus tantalus* -....**A**.......**A**....**C**.....................................................**T**.**G**.......**A**......**A**................**G**...

*Chlorocebus pygerythrus* -....**A**.......**A**....**C**.....................................................**T**.**G**.......**A**......**A**.........**G**......**G**...

*Cercopithecus diana* -....**A**.......**A**....**C**.....................................................**T**.**G**.......**A**......**A**................**G**...

*Cercocebus torquatus* -....**A**.......**A**....**C**.....................................................**T**.**G**.......**A**......**A**................**G**...

*Papio hamadrayas*  -....**A**.......**A**....**C**.....................................................**T**.**G**.......**A**......**A**................**G**...

*Macaca mulatta*  -....**A**.......**A**....**C**.................**A**...................................**T**.**G**.......**A**......**A**................**G**...

*Ateles geoffroyi*  **C**..................................**C**...........**GG**-.....**T**.-................**G**.......**C**......**A**................**G**...

*Lagothrix lagotricha*  **C**..................................**C**...........**GG**-.....**T**.-................**G**.......**.**......**A**................**G**...

*Cebus capucinus*  **C**.............................**C**....**C**.......**A**...**GG**-........................**G**.......**.A**.....**A**............**T**...**G**...

*Saimiri sciureus*  **C**................**G**.................**C**...........**GG**-...........**T**............**GC**......**.**......**A**................**G**...

*Aotus trivirgatus*  -..................................**C**...........**GG**-........................**G**.......**.**......**A**................**G**...

**** **** ** *** ** * ** ** **** ** *** *** * ** * *** ** ****** * ** ** ***** ********* ** ** ***

upSp1 NFAT NFAT/ETS CRE κ3 ETS

-189 -100 -80

*Homo sapiens* TCCCCAACTTTCCAAAT**CCCCGCCCCC**GCGATG**GAGAAGAAA**CCGAGACAGAAGGTGCAGGGCCCACTACCG**CTTCC**TCCAGA**TGAGCTCA**T**GGGTTTCTCC**ACC**AAGGA**

*Pan paniscus*  ..............................................................................................................

*Pan troglodytes verus*  ..............................................................................................................

*Pan t. troglodytes*  ..............................................................................................................

*Pan t. vellerosus*  ..............................................................................................................

*Pan t. schweinfurthii*  ..............................................................................................................

*Gorilla gorilla gorilla* ..............................................................................................................

*Gorilla beringei graueri*..............................................................................................................

*Gorilla b. beringei* ..............................................................................................................

*Pongo pygmaeus* .....................**A**......**T**.................................................................................

*Pongo abelii*  .....................**A**......**T**.................................................................................

*Hylobates lar*  .........................**T**....................................................................................

*Hylobates moloch*  ............................**T**.................................................................................

*Hylobates agilis*  ............................**T**.................................................................................

*Hylobates pileatus*  ............................**T**.................................................................................

*Hoolock leuconedys*  .........................**T**....................................................................................

*Nomascus l. leucogenys*  .....................**A**...**T**....................................................................................

*Symphalangus syndactylus*.........................**T**....................................................................................

*Chlorocebus sabaeus* ............................................**A**...**G**.............................................................

*Chlorocebus tantalus* ............................................**A**...**G**.............................................................

*Chlorocebus pygerythrus* ............................................**A**...**G**...................................................**T.........**

*Cercopithecus diana* ............................................**A**.................................................................

*Cercocebus torquatus* ............................................**A**.................................................................

*Papio hamadrayas*  ............................................**A**.................................................................

*Macaca mulatta*  ............................................**A**.................................................................

*Ateles geoffroyi*  ..................**T**..**A**.....................**G**.....**T**......**TT**....................................................

*Lagothrix lagotricha*  ..................**T**..**A**.....................**G**.....**T**....**A**.**TT**....................................................

*Cebus capucinus*  ..................**T**..**A**......**T**..............**G**.....**T**....**C**.**TT**.................................**A..................**

*Saimiri sciureus*  ..................**T**..**A**......**T**..............**A**.....**T**....**C**.**T**.....................................................

*Aotus trivirgatus*  ..................**T**..**A**.....................**G**.....**T**......**TT**....................................................

****************** ** *** ** ************** *** **** * ********************************* ******** *********

NFAT NFAT/Sp1

-79 -9 +1 +30

*Homo sapiens* **A**G**TTTTCC**GCTGGTTGAATGATTC**TTTCCCCGCCC**TCCTCTCGCCCCAGGGACA-TATAAAGGCAGTTGTT**G**GCACACCC**A**GCCAGCAGACGCTCCCTCAGCAAGGACAG

*Pan paniscus*  ......................................................-................**T**......................................

*Pan troglodytes verus*  ......................................................-................**T**......................................

*Pan t. troglodytes*  ......................................................-................**T**......................................

*Pan t. vellerosus*  ......................................................-................**T**......................................

*Pan t. schweinfurthii*  ......................................................-................**T**......................................

*Gorilla gorilla gorilla* ......................................................-................**T**......................................

*Gorilla beringei graueri*......................................................-................**T**......................................

*Gorilla b. beringei* ......................................................-................**T**......................................

*Pongo pygmaeus* ......................................................-................**T**......................................

*Pongo abelii*  ......................................................-................**T**......................................

*Hylobates lar*  ......................................................-................**T**......................................

*Hylobates moloch*  ......................................................-................**T**......................................

*Hylobates agilis*  ......................................................-................**T**......................................

*Hylobates pileatus*  ......................................................-................**T**......................................

*Hoolock leuconedys*  ...............----...................................-................**T**......................................

*Nomascus l. leucogenys*  ......................................................-................**T**......................................

*Symphalangus syndactylus*......................................................-................**T**......................................

*Chlorocebus sabaeus* ...................................---................-......**C**.........**T**......................................

*Chlorocebus tantalus* ...................................---................-......**C**.........**T**......................................

*Chlorocebus pygerythrus* ...................................---................-......**C**.........**T**......................................

*Cercopithecus diana* ...................................---................-......**C**.........**T**......................................

*Cercocebus torquatus*  .........................**A**.........---................-......**C**.........**T**......................................

*Papio hamadrayas*  .........................**A**.........---................-......**C**.........**T**......................................

*Macaca mulatta*  .........................**A**.........---................-......**C**.........**T**......................................

*Ateles geoffroyi*  .................**T**........................**C**..**T**.......**TT**................**T**-................................**A**....

*Lagothrix lagotricha*  .................**T**........................**C**..**T**.......**TT**................**T**-................................**A**....

*Cebus capucinus*  .................**T**........................**C**..**T**.......**TT**................**T**-................................**A**....

*Saimiri sciureus*  .................**T**..................**T**.....**C**..**T**.......**TT**................**T**-.....................................

*Aotus trivirgatus*  .................**T**...................**T**....**C**..**T**.......**TT**................**T**-.....**T**...........**A**...................

*************** ****** ********* **** ** ******* ****** ********* ***** *********** ************** ****

+31 +69

*Homo sapiens* CAGAGGACCAGCTAAGAGGGAGAGAAGCAACTACAGACC

*Pan paniscus*  .......................................

*Pan troglodytes verus*  .......................................

*Pan t. troglodytes*  .......................................

*Pan t. vellerosus*  .......................................

*Pan t. schweinfurthii*  .......................................

*Gorilla gorilla gorilla* .......................................

*Gorilla beringei graueri*.......................................

*Gorilla b. beringei* .......................................

*Pongo pygmaeus* .......................................

*Pongo abelii*  .......................................

*Hylobates lar*  .......................................

*Hylobates moloch*  .......................................

*Hylobates agilis*  .......................................

*Hylobates pileatus*  .......................................

*Hoolock leuconedys*  .......................................

*Nomascus l. leucogenys*  .......................................

*Symphalangus syndactylus*.......................................

*Chlorocebus sabaeus* ................................**C**......

*Chlorocebus tantalus* ................................**C**......

*Chlorocebus pygerythrus* ................................**C**......

*Cercopithecus diana* ................................**C**......

*Cercocebus torquatus* ................................**C**......

*Papio hamadrayas*  ..........................**A**.....**C**......

Macacamula ................................**C**......

*Ateles geoffroyi*  ............**C**.................**T**.**C**......

*Lagothrix lagotricha*  ............**C**.................**T**.**C**......

*Cebus capucinus*  ............**C**.................**T**.**C**......

*Saimiri sciureus*  ............**C**.................**T**.**C**....**T**.

*Aotus trivirgatus*  ............**C**....**A**............**T**.**T**......

************ **** ******** *** * **** *
