# Supplementary material for: Primate TNF Promoters Reveal Markers of Phylogeny and Evolution of Innate Immunity
Source: PLoS One. 2007 Jul 18;2(7):e621. doi: 10.1371/journal.pone.0000621 (PMC1905939; doi:10.1371/journal.pone.0000621)
Supplement: Table S1 — Total fixed differences among the primate TNF promoters. Positions of single nucleotide changes and discrete insertions or deletions, relative to the consensus human TNF promoter sequence, observed in this study are shown. (0.04 MB PDF) [file pone.0000621.s002.pdf]

Position	Fixed difference
+68	C/T
+63	A/C
+63	A/T
+61	C/T
+57	G/A
+48	G/A
+43	T/C
+26	G/A
+11	C/A
-2	C/T
-8	ΔG
-9	G/T
-19	G/C
-25	+T
-26	A/T
-34	C/T
-37	G/C
-42	C/T
-42 to -44	ΔTCC
-43	C/T
-54	T/A
-61 to -64	ΔGAAT
-62	A/T
-89	C/T
-98	T/A
-132	C/T
-133	G/T
-135	G/C
-135	G/A
-140	A/T
-141	C/G
-145	G/A
-146	C/A
-146	C/G
-161	C/T
-164	C/T
-168	G/A
-171	C/T
-193	T/G
-194	G/A
-197	C/T
-200	A/G
-210	G/A
-216	G/A
-217	T/A
-217	T/C
-218	G/C
-221	G/C
-224	T/C
-225	A/G
-227	G/T
-234	G/A
-237	G/A
-238	C/T
-242	ΔG
-244	C/T
-247	C/T
-249	C/T
-250	ΔC
-251	C/G
-252	A/G

Position	Fixed difference
-256	G/A
-260	C/A
-263	T/A
-264	G/C
-269	A/C
-272	A/G
-275	C/T
-277	C/T
-280	A/G
-281	T/C
-282	C/G
-286	G/A
-289	C/T
-294	G/A
-298	+AC
-299	G/A
-299 to -301	ΔGGG
-301	G/A
-302	G/A
-302	G/C
-303	C/A
-304	A/G
-305	G/C
-307	G/A
-309	T/G
-321	T/C
-325	T/C
-329	G/A
-332	G/C
-333	G/A
-333	G/T
-334	T/C
-335	A/C
-341	A/G
-356	C/T
-369	A/G
-372	A/G
-373	T/C
-375	G/A
-376	A/C
-377	A/G
-378	G/A
-379	G/A
-395	T/C
-402 to -399	ΔTATC
-400	T/C
-405	T/C
-406	G/C
-407	A/C
-412	G/T
-418	C/T
-431	C/G
-431	C/T
-441	A/G
-442	C/T
-443	T/C
-444	C/A
-445	C/T
-446	C/A
-448	C/G

-455	G/A
-456	C/T
-458	C/T
-462	C/T
-467	+T
-467	+C
-470	T/A
-472	C/A
-474	A/G
-477	C/A
-491	G/A
-492	G/A
-495	A/G
-500	C/A
-504	G/A
-508	C/A
-509	G/C
-510	C/T
-511	C/T
-512	C/T
-513	C/T
-515	C/T
-518	C/T
-521	C/T
-522	G/A
-523	A/G
-524	C/T
-527	+CTGGAGCTGCCCTGT
-527	+ATGGAGCTGCCCTGT
-528	C/T
-530	A/C
-530	A/G
-534	A/C
-536	C/T
-539	C/T
-540	C/T
-540	C/G
-541	G/A
-541	G/C
-543	A/G
-544	C/T
-545	T/C
-547	C/T
-550	+T
-552	T/C
-555	T/C
-556	T/A
-559	C/T
-564	C/T
-566	C/T
-567	C/T
-581	T/C
-597	G/A
-598	C/T
-612	G/C
-615	G/A
-616	G/A
-627	T/C
-637	C/T
-637	ΔC
-638	C/T
-640	C/T
-641	G/A
-645 to -641	ΔGCCTG
-645	G/C
-647	A/G
-647	A/T
-648	A/T
-649	G/A
-651	A/G
-652	G/A
-658	A/G
-658	A/C
-660	G/A
-660	G/T
-662	C/A
-666	G/C
-667	C/T
-667	C/A
-667	C/G
-669	T/C
-676	A/G
-678	T/G
-705	C/G
-705	C/T
-709	A/C
-710	G/C
-710	G/A
-723	ΔA
-724	A/T
-727	A/C
-730	G/A
-731	T/C
-736	C/T
-743	G/A
-750	C/A
-750	C/G
-751	C/T
-751	C/G
-754	G/A
-758	C/T
-761	A/G
-770	T/C
-771	A/C
-777	A/C
-781	G/C
-781	G/A
-782	C/T
-787	G/C
-790	+AAT
-795	G/C
-796	T/C
-802	C/T
-815	C/T
-823	T/C
-827	G/A
-830	C/T
-840	T/C
-841	G/A
-846	G/A
-848	G/A
-855	G/A
-855	G/C
-857	A/G
-857	ΔA
-858	ΔA

-859	ΔT
-859	T/C
-860	ΔT
-860	T/C
-860	T/G
-861	ΔC
-861	C/T
-862	C/G
-862	C/A
-863	C/T
-864	C/T
-865	C/T
-869	G/T
-874	A/G
-879	C/T
-879	C/G
-882	A/G
-883	A/G
-887	A/G
-890	G/A
-891	G/A
-893	A/G
-894	C/T
-898	T/A
-909	G/A
-910	T/A
-915	A/G
-916	C/T
-923	G/A
-928	G/A
-938	G/C
-943	C/A
-945	T/G
-962	G/A
-963	C/T
-968	T/A
-973	C/T
-976	T/C
-979	A/G
-980	+GG
-981	A/G
-985	T/C
-987	C/G
-988	T/C
-1002	G/A
-1003	C/A
-1009	G/A
-1009	G/C
-1014	G/A
-1020	G/A
-1022 to -1027	ΔAGGAAA
-1029	G/A
-1030	T/A
-1030	T/C
-1031	A/G
-1035	G/T
-1042	A/G
-1043	G/T
-1064	T/G
-1066	A/G
-1069	C/T
-1072	C/T
-1072	C/A
-1082	C/G
-1084	A/G
-1088	A/G
-1091	T/C
-1094	T/C
-1098	T/C
-1102	G/A
-1107	C/T
-1111	A/G
-1112	G/T
-1119	G/T
-1119	G/A
-1119	+A
-1123	A/G
-1126	A/G
-1127	A/G
-1129	A/G
-1135	G/C
-1137	G/A
-1145	G/C
-1148	C/G
