# Supplementary material for: Primate TNF Promoters Reveal Markers of Phylogeny and Evolution of Innate Immunity
Source: PLoS One. 2007 Jul 18;2(7):e621. doi: 10.1371/journal.pone.0000621 (PMC1905939; doi:10.1371/journal.pone.0000621)
Supplement: Table S2 — SNPs in primate TNF promoters. For each of the indicated species or subspecies, the position, sequence change, and frequency (in the total number of individuals examined) of each SNP detected in the TNF promoter is shown. SNPs in which the wild type allele corresponds to the sequence found in the other subspecies of a given species are shown, while the mutant alleles that correspond to the human sequence are noted with an asterisk. (0.07 MB PDF) [file pone.0000621.s003.pdf]

Species	Position	Common/Variant Allele	Frequency
Pan paniscus	-856	C/T	3/7
Pan troglodytes troglodytes	-1027 to -1029	GAA/ΔΔΔ	2/5
	-799	G/A	1/5
	-472	C/T	1/5
	-327	A/G	1/5
Pan troglodytes vellerosus	-799	G/A	1/3
Pan troglodytes schweinfurthii	-778	T/C	1/2
Chlorocebus sabaeus	-614	T/G*	1/4
	-322	G/A	2/4
Chlorocebus tantalus	-879	T/C*	1/6
	-857	G/A*	1/6
	-756	G/A	1/6
	-352	C/T	3/6
Chlorocebus pygerythrus	-919	C/A	2/3
	-850	C/G	2/3
	-809	A/G*	1/3
	-756	G/A	2/3
	-352	C/T	1/3
	-303	C/T	1/3
