# Supplementary material for: Primate TNF Promoters Reveal Markers of Phylogeny and Evolution of Innate Immunity
Source: PLoS One. 2007 Jul 18;2(7):e621. doi: 10.1371/journal.pone.0000621 (PMC1905939; doi:10.1371/journal.pone.0000621)
Supplement: Table S3 — Fixed differences in the TNF promoter that mark primate clades. Total fixed genetic differences in the TNF promoter that are unique to and that are completely conserved within the indicated primate taxa are shown. A. For each position, the sequence found within and outside of the indicated clades is shown. B. For each position, the sequence found in humans and in the indicated genus or clade is shown. The Cebidae family includes Cebus capucinus and Saimiri sciureus; the Atelinae subfamily, Ateles geoffroyi and Lagothrix lagotricha. See Figure 6 for diagram of evolutionary context. (0.03 MB PDF) [file pone.0000621.s004.pdf]

A.

Position clade allele/non-clade allele

Human+African great ape specific:

Position	clade allele/non-clade allele
-225	A/G
-509	G/C
-543	A/G
-555	T/C
-676	A/G
-882	A/G
-910	T/A
-963	C/T
-1119	-/A
-1029	G/A

Human+ape specific:

Position	clade allele/non-clade allele
-193	T/G
-210	G/A
-321	T/C
-369	A/G
-373	T/C
-375	G/A
-534	A/C
-545	T/C
-669	T/C
-855	G/C
-858	A/-
-883	A/G

Human+ape+OWM specific:

Position	clade allele/non-clade allele
+61	C/T
+43	T/C
-8	G/-
-25	-/T
-26	A/T
-34	C/T
-37	G/C
-62	A/T
-140	A/T
-171	C/T
-250	C/-
-251	C/G
-252	A/G
-303	C/A
-304	A/G
-329	G/A
-332	G/C
-333	G/T
-376	A/C
-406	G/C
-407	A/C
-441	A/G
-442	C/T
-458	C/T
-511	C/T
-523	A/G
-524	C/T
-528	C/T
-547	C/T
-556	T/A
-598	C/T
-612	G/C
-651	A/G
-678	T/G
-705	C/T
-723	A/-
-727	A/C
-743	G/A
-782	C/T
-840	T/C
-841	G/A
-874	A/G
-945	T/G
-973	C/T
-1072	C/A
-1088	A/G
-1127	A/G
-1135	G/C
-1145	G/C

B.

Position	human allele/non-human allele
***Gorilla* specific:**	
-724	A/T
-943	C/A
-962	G/A
***Pongo* specific:**	
-194	G/A
-275	C/T
-277	C/T
-333	G/A
-500	C/A
-522	G/A
-777	A/C
-787	G/C
-848	G/A
-1082	C/G
**small ape specific:**	
-289	C/T
-431	C/G
-637	C/T
-859	T/C
-860	T/C
-1064	T/G
-1072	C/T
**OWM specific:**	
-19	G/C
-42 to -44	TCC/---
-145	G/A
-217	T/A
-227	G/T
-281	T/C
-286	G/A
-294	G/A
-307	G/A
-309	T/G
-446	C/A
-467	+CT
-504	G/A
-508	C/A
-736	C/T
-802	C/T
-830	C/T
-968	T/A
-1009	G/A
-1098	T/C
